# Glutathione limits RUNX2 oxidation and degradation to regulate bone formation

**DOI:** 10.1172/jci.insight.166888

**Published:** 2023-08-22

**Authors:** Guoli Hu, Yilin Yu, Deepika Sharma, Shondra M. Pruett-Miller, Yinshi Ren, Guo-Fang Zhang, Courtney M. Karner

**Affiliations:** 1 Department of Internal Medicine, University of Texas Southwestern Medical Center, Dallas, Texas, USA.; 2 Department of Orthopaedic Surgery, Duke University School of Medicine, Durham, North Carolina, USA.; 3 Department of Cell and Molecular Biology, St. Jude Children’s Research Hospital, Memphis, Tennessee, USA.; 4 Center for Excellence in Hip Disorders, Texas Scottish Rite Hospital for Children, Dallas, Texas, USA.; 5 Department of Medicine, Division of Endocrinology, Metabolism Nutrition, and; 6 Sarah W. Stedman Nutrition and Metabolism Center & Duke Molecular Physiology Institute, Duke University, Durham, North Carolina, USA.; 7 Charles and Jane Pak Center for Mineral Metabolism and Clinical Research, University of Texas Southwestern Medical Center, Dallas, Texas, USA.

**Keywords:** Bone Biology, Amino acid metabolism, Bone development, Osteoclast/osteoblast biology

## Abstract

Reactive oxygen species (ROS) are natural products of mitochondrial oxidative metabolism and oxidative protein folding. ROS levels must be well controlled, since elevated ROS has been shown to have deleterious effects on osteoblasts. Moreover, excessive ROS is thought to underlie many of the skeletal phenotypes associated with aging and sex steroid deficiency in mice and humans. The mechanisms by which osteoblasts regulate ROS and how ROS inhibits osteoblasts are not well understood. Here, we demonstrate that de novo glutathione (GSH) biosynthesis is essential in neutralizing ROS and establish a proosteogenic reduction and oxidation reaction (REDOX) environment. Using a multifaceted approach, we demonstrate that reducing GSH biosynthesis led to acute degradation of RUNX2, impaired osteoblast differentiation, and reduced bone formation. Conversely, reducing ROS using catalase enhanced RUNX2 stability and promoted osteoblast differentiation and bone formation when GSH biosynthesis was limited. Highlighting the therapeutic implications of these findings, in utero antioxidant therapy stabilized RUNX2 and improved bone development in the *Runx2^+/–^* haplo-insufficient mouse model of human cleidocranial dysplasia. Thus, our data establish RUNX2 as a molecular sensor of the osteoblast REDOX environment and mechanistically clarify how ROS negatively impacts osteoblast differentiation and bone formation.

## Introduction

Osteoblasts are the exclusive bone-forming cells that synthesize and secrete the collagen type I α-1–rich (COL1A1-rich) extracellular bone matrix (1, 2). Commitment to the osteoblast lineage and osteoblast differentiation are tightly regulated by the transcription factor RUNX2. Autosomal-dominant loss-of-function mutations in RUNX2 cause the disease cleidocranial dysplasia (CCD), which is characterized by patent fontanelles and hypoplastic clavicles (3, 4). Highlighting the importance of tight regulation of RUNX2 expression and activity, CCD has been proposed to arise due to relatively minor reductions in RUNX2 dosage (5). RUNX2 regulates osteoblast commitment and differentiation through direct transcriptional regulation of osteoblast genes (e.g., *Sp7*, *Col1a1*, and *Bglap*) as well as through favoring glucose consumption (3, 6–11). The high anabolic and biosynthetic activity associated with bone formation burdens osteoblasts with increased energetic and biosynthetic demands. To meet these demands, osteoblasts increase the consumption of nutrients like glucose, glutamine, and proline and increase ATP production via glycolysis and mitochondrial oxidative phosphorylation (OXPHOS) (12–19). When nutrients are limited, osteoblast differentiation and bone formation fail. For example, genetic mutations that limit glutamine or proline uptake reduce the synthesis of RUNX2 and COL1A1 (20, 21). Conversely, genetically limiting glucose uptake reduces ATP levels and secondarily stimulates RUNX2 degradation, highlighting the intimate link between nutrient availability, metabolism, and the regulation of osteoblast differentiation by RUNX2 (10).

The endoplasmic reticulum and mitochondria are 2 primary sources of reactive oxygen species (ROS) (22, 23). Due to increased electron-transport chain (ETC) activity and oxidative protein folding in the ER, osteoblast differentiation is predicted to result in increased ROS generation (24, 25). At physiological levels, ROS is an important signaling molecule, but when elevated, ROS can have deleterious effects on osteoblasts, preventing differentiation and, in severe cases, resulting in senescence and apoptosis (26–29). Elevated ROS underlies many of the skeletal phenotypes observed in aging and in response to sex steroid deficiency in mice and humans (30–32). The precise mechanisms by which ROS limits osteoblast differentiation remains unclear.

**Figure 1:**
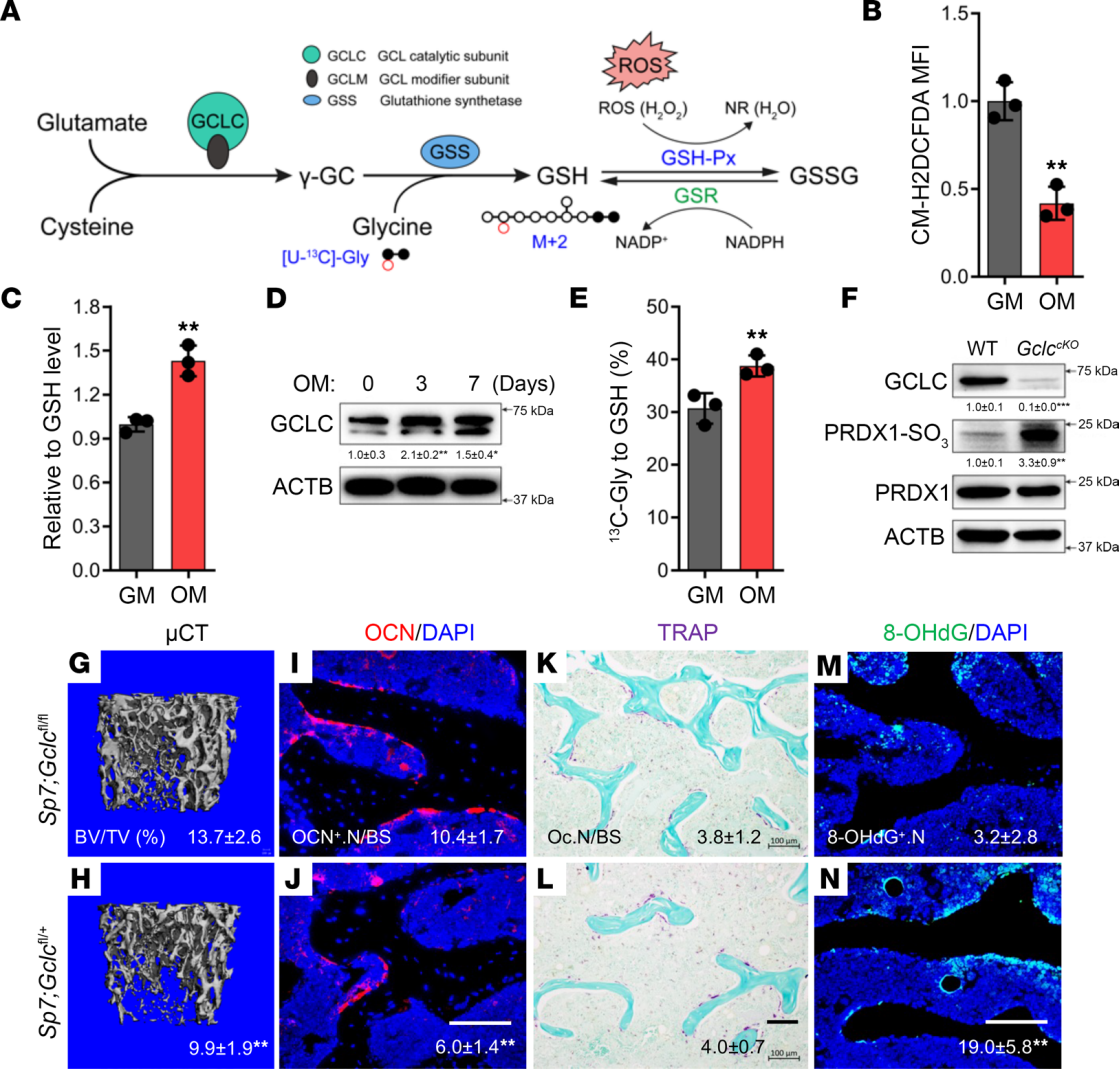
GSH biosynthesis is essential for osteoblast differentiation and bone formation. (**A**) Schematic showing GSH biosynthesis pathway and [U-^13^C]glycine tracing. Black filled circles indicate ^13^C, whereas black open circles denote ^12^C. γ-GC, γ-glutamylcysteine; GSSG, glutathione disulfide; GSR, glutathione reductase; GSH-Px, glutathione peroxidase. (**B**) Flow cytometry analysis of ROS levels in naive and differentiated calvarial cells (*n* = 3). GM, growth media; OM, osteogenic media. (**C**) Relative intracellular GSH levels in naive and differentiated calvarial cells measured by mass spectrometry (*n* = 3). (**D**) Western blot analysis of GCLC (normalized to ACTB) (*n* = 3). (**E**) Fractional contribution of [U-^13^C]glycine to GSH measured by mass spectrometry (*n* = 3). (**F**) Western blot analysis of GCLC, PRDX1-SO_3_, and PRDX1 in bone extracts isolated from *Sp7Cre;Gclc^fl/+^* or *Sp7Cre;Gclc^fl/fl^* mice (*n* = 4). PRDX1-SO_3_ was normalized to total PRDX1; GCLC was normalized to ACTB. (**G** and **H**) Representative μCT images of trabecular bone from distal femur of 6-month-old mice (*n* = 8). BV/TV is listed below each image. (**I**–**N**) Representative histological sections showing osteocalcin (OCN) immunofluorescence (**I** and **J**), TRAP staining (**K** and **L**), and 8-OHdG immunofluorescence (**M** and **N**) performed in distal femurs from *Sp7Cre;Gclc^fl/+^* and *Sp7Cre;Gclc^fl/fl^* mice (*n* = 8). Scale bar: 100 μm. Quantifications of the individual stains are listed below each image. Data are shown as mean ± SD. **P < 0.01. Two-tailed Student’s unpaired *t* test (**B**, **C**, and **E**), 1-way ANOVA (**D**), and 2-tailed Student’s paired *t* test (**F**–**N**) were used.

ROS must be tightly controlled for osteoblast differentiation to proceed. Osteoblasts rely on superoxide dismutase (SOD) to convert reactive O_2_^–^ into H_2_O_2_ (33–35). H_2_O_2_ can be detoxified to water by several enzymes, including the glutathione (GSH) peroxidases (GPXs), which use GSH as the reducing agent (36). GSH is a tripeptide composed of glutamate, cysteine, and glycine that is synthesized de novo (37–39). The enzyme glutamate-cysteine ligase (GCL, a heterodimer composed of a catalytic [GCLC] and modifier [GCLM] subunits) catalyzes the first rate-limiting step of GSH biosynthesis, the conjugation of cysteine and glutamate to form γ-glutamylcysteine (γ-GC). γ-GC is then ligated to glycine by GSH synthase (GS) to form GSH (Figure 1A) (40–43). GSH biosynthesis is determined by the availability of its constituent amino acids, which intersects with various metabolic pathways. For example, glutamine metabolism provides glutamate, which can be incorporated into GSH but can also regulate cysteine uptake through solute carrier family 7 member 11 (SLC7A11, also called xCT) (44, 45). Inhibiting glutamine metabolism by deleting the enzyme glutaminase (GLS) in osteoblasts or their progenitors results in decreased bone mass and is associated with reduced GSH levels (46, 47). Pharmacologically reducing GSH levels in vitro using buthionine sulfoximine (BSO) reduces osteoblast viability. However, BSO treatment increased osteoblast numbers in vivo despite reducing overall bone mass (48). Thus, the role and requirement for de novo GSH biosynthesis during osteoblast differentiation and bone formation remains to be elucidated.

In the present study, we define the role of GSH biosynthesis to establish a proosteogenic reduction and oxidation reaction (REDOX) environment essential for osteoblast differentiation and bone formation. Using metabolomic and genetic approaches, we demonstrate that differentiating osteoblasts increase GSH biosynthesis. Genetically limiting GSH biosynthesis resulted in DNA and protein oxidation highlighted by a reduction of RUNX2. Mechanistically, ROS directly oxidizes RUNX2, resulting in proteasomal degradation and inhibition of osteoblast differentiation. Importantly, reducing ROS by ectopic expression of catalase (CAT) stabilizes RUNX2 protein and promotes bone formation when GSH biosynthesis is genetically limited in vivo. Collectively, these data highlight the importance of tight REDOX regulation in osteoblasts due to the sensitivity of RUNX2 to ROS.

## Results

### GSH biosynthesis increases and is required for osteoblast differentiation and bone formation.

Osteoblast differentiation is associated with increased protein synthesis and OXPHOS, both primary sources of ROS (1, 2, 12, 13, 15). Despite this, total ROS declined significantly during osteoblast differentiation (Figure 1B). Concomitant with decreased ROS, differentiated osteoblasts were characterized by significantly higher GSH compared with naive calvarial cells (Figure 1C). Moreover, osteoblasts had higher mRNA or protein expression of several enzymes involved in either GSH biosynthesis (e.g., *Gclc*, *Gclm*, and *Gss*) or GSH-dependent ROS neutralization (e.g., *Gpx3*, *Gst*, and *Gsr*) compared with naive calvarial cells (Figure 1D and Supplemental Figure 1A; supplemental material available online with this article; https://doi.org/10.1172/jci.insight.166888DS1). Consistent with the gene expression studies, stable isotopomer analysis found that [U-^13^C]glycine incorporation into GSH increased significantly in differentiated osteoblasts (Figure 1E). Thus, increased GSH biosynthesis is a hallmark of osteoblast differentiation.

We next sought to determine the necessity of GSH biosynthesis for osteoblast differentiation. To do this, we first depleted intracellular GSH using 4 distinct strategies: pharmacological inhibition of GCLC using BSO, genetic ablation of either *Gclc* or *Gclm* using CRISPR/Cas9 targeting, or inhibition of the cysteine transporter xCT using Erastin to limit cysteine availability (Supplemental Figure 1, B–F). All 4 strategies depleted intracellular GSH and significantly increased ROS accumulation (Supplemental Figure 1, G and H). Consistent with increased ROS, GCLC, or xCT inhibition resulted in protein oxidation (exemplified by peroxiredoxin 1 sulfonation [PRDX1-SO_3_]) and increased expression of oxidative stress responsive proteins (e.g., NAD[P]H quinone dehydrogenase 1 [NQO1]) (Supplemental Figure 1, J and K). Moreover, reducing intracellular GSH prevented osteoblast differentiation and matrix mineralization, regardless of the method used (Supplemental Figure 1, I, L, and M). Thus, the maintenance of intracellular GSH is essential for osteoblast differentiation in vitro.

We next sought to determine if GSH biosynthesis is required for osteoblast differentiation and bone formation in vivo. To do this, we deleted a conditionally null (floxed) allele of *Gclc* (*Gclc^fl^*) in specified preosteoblasts using the *Sp7-tTA;tetO-EGFP/Cre* mouse (referred to here as *Sp7Cre*). CRE expression was inhibited using doxycycline until weaning, and bone phenotypes were analyzed at 6 months of age. This approach efficiently ablated GCLC protein, as determined by Western blot (Figure 1F). *Gclc* deletion resulted in reduced bone mass in *Sp7Cre;Gclc^fl/fl^* mice, as determined by μCT (Figure 1, G and H, and Supplemental Table 1). Quantification of the μCT data found that the trabecular bone volume (BV/TV) and bone mineral density (BMD) were significantly reduced in both male and female *Sp7Cre;Gclc^fl/fl^* animals (Supplemental Table 1). Additionally, *Sp7Cre;Gclc^fl/fl^* bones were characterized by decreased trabecular number (Tb.N) and trabecular thickness (Tb.Th) with increased trabecular separation (Tb.Sp) (Supplemental Table 1). *Gclc* deletion did not affect cortical bone thickness (Ct.Th) or total bone area (T.Ar) compared with *Sp7Cre*^+^ WT controls (Supplemental Table 1). Decreased bone mass was attributed to defects in osteoblasts and bone formation, as there were significantly fewer osteocalcin^+^ (OCN^+^) osteoblasts on the bone surface with no difference in the number of TRAP^+^ osteoclasts in *Sp7Cre;Gclc^fl/fl^* mice compared with CRE^+^ WT littermates (Figure 1, I–L, and Supplemental Table 1). *Gclc* ablation also impaired bone-forming activity as both the mineral apposition rate (MAR) and bone formation rate (BFR) were significantly reduced in the *Sp7Cre;Gclc^fl/fl^* mice compared with *Sp7Cre;Gclc^fl/+^* littermates (Supplemental Table 1). Likewise, serum levels of OCN and N-terminal propeptide of collagen type I (P1NP), markers of bone formation, were significantly reduced in *Sp7Cre;Gclc^fl/fl^* mice (Supplemental Table 1). Serum levels of the C-terminal telopeptide of collagen (CTX-1), a marker of bone resorption, was not changed (Supplemental Table 1). *Sp7Cre;Gclc^fl/fl^* bones were characterized by extensive protein and DNA oxidation (exemplified by PRDX1-SO_3_ and 8-hydroxy-2′-deoxyguanosine [8-OHdG], respectively) (Figure 1, F, M, and N). In general, the oxidative stress caused by loss of GCLC was relatively mild, as we did not observe double-stranded DNA breaks (e.g., no TUNEL^+^ cells) or increased expression of markers of either apoptosis (e.g., cleaved caspase-3) or senescence in either bones or primary osteoblasts from *Sp7Cre;Gclc^fl/fl^* mice (Supplemental Figure 2). Taken together, these data indicate that de novo GSH biosynthesis is critical for the neutralization of ROS generated during osteoblast differentiation and bone formation.

### RUNX2 is directly oxidized by H_2_O_2_.

**Figure 2:**
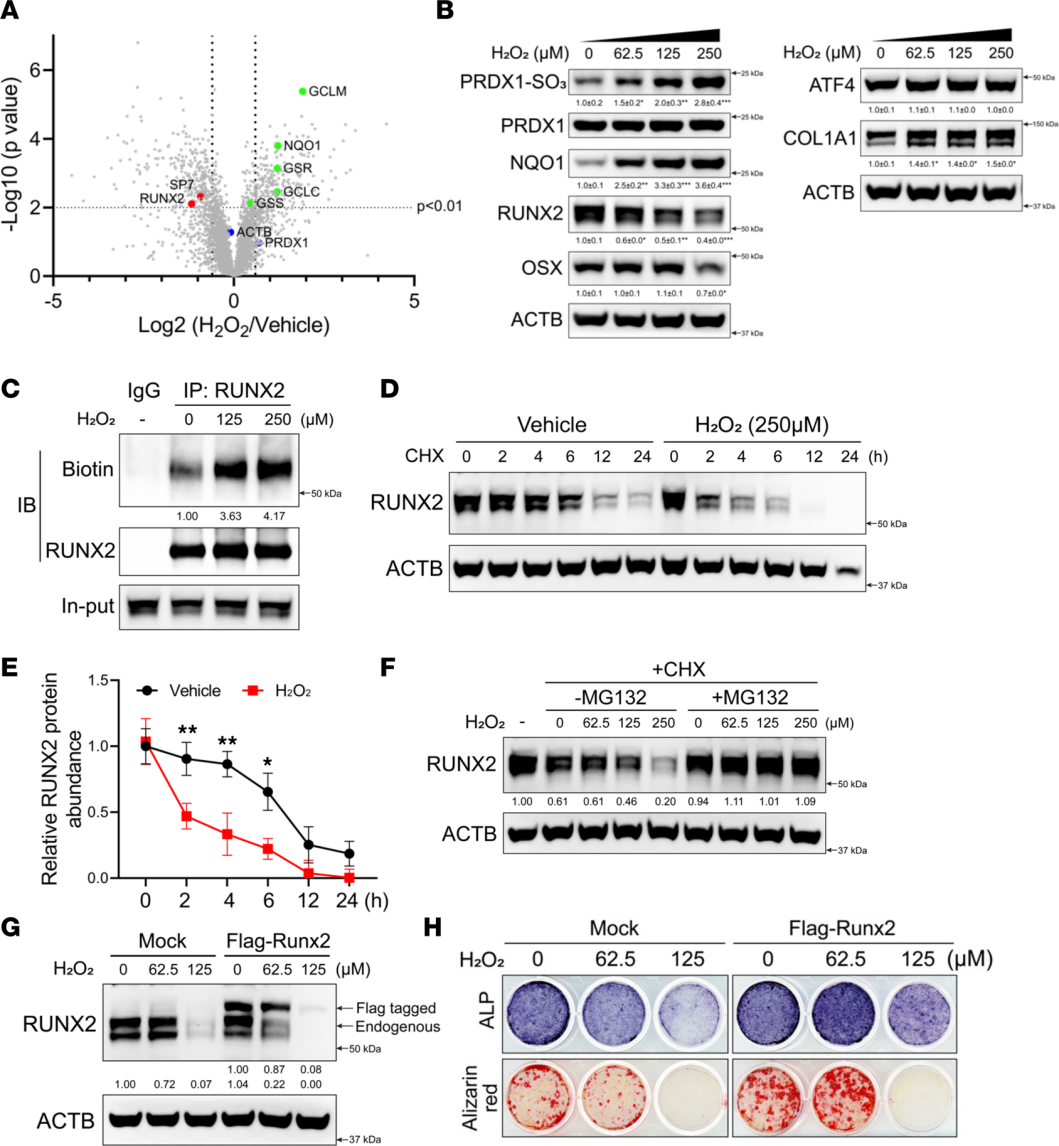
ROS inhibits osteoblast differentiation by oxidizing and degrading RUNX2. (**A**) Volcano plot showing the graphical depiction of protein expression in H_2_O_2_-treated calvarial cells (*n* = 3). (**B**) Western blot analysis of PRDX1-SO_3_ (normalized to total PRDX1), NQO1, RUNX2, OSX, ATF4, and COL1A1 in naive calvarial cells treated with a gradient of H_2_O_2_ (*n* = 3). ATF4 and COL1A1 were from a separate experiment (*n* = 3). Total protein normalized to ACTB. (**C**) Western blot analysis of immunoprecipitated RUNX2 from H_2_O_2_-treated calvarial cells (*n* = 3). Normal rabbit IgG was used as a negative control. (**D** and **E**) Western blot analysis of the effect of H_2_O_2_ on RUNX2 degradation in calvarial cells (*n* = 3). Quantification of 3 independent experiments is shown in **E**. (**F**) Western blot analysis of the effect of MG132 on RUNX2 expression in calvarial cells treated with H_2_O_2_ for 4 hours (*n* = 3). (**G**) Western blot analysis of RUNX2 in calvarial cells transfected with or without FLAG-RUNX2 under different doses of H_2_O_2_ (*n* = 3). (**H**) ALP and alizarin red staining in calvarial cells transfected with or without FLAG-RUNX2 under different doses of H_2_O_2_ (*n* = 3). Data are shown as mean ± SD. *P < 0.05, **P < 0.01. Two-tailed Student’s unpaired *t* test.

We next sought to investigate the molecular effects of elevated ROS on osteoblasts. H_2_O_2_ treatment increased intracellular ROS and inhibited osteoblast differentiation in a dose-dependent manner reminiscent of GCLC inhibition (Supplemental Figure 3, A and B). To understand the molecular basis for this, we characterized the effects of H_2_O_2_ on the osteoblast proteome in naive calvarial cells. H_2_O_2_ increased the expression of the oxidative stress–sensitive protein NQO1 and the GSH biosynthetic enzymes (e.g., GCLC, GCLM, GSS, and GSR) (Figure 2A). Conversely, several osteoblast proteins, including the transcription factors RUNX2 and OSX, were reduced (Figure 2A). Western blot analyses confirmed that H_2_O_2_ treatment increased NQO1 expression and increased protein oxidation, as evidenced by PRDX1-SO_3_ (Figure 2B). Moreover, H_2_O_2_ reduced RUNX2 and OSX protein expression in a dose-dependent manner without affecting the expression of ATF4 and increased COL1A1 expression (Figure 2B). Increased COL1A1 protein is likely due to reduced expression of several members of the COPII-dependent collagen secretory pathway, including SAR1, SEC13, and SEC31, which were reduced by H_2_O_2_ treatment (49–51). RUNX2 was reduced at all concentrations of H_2_O_2_ (Figure 2B). By comparison, OSX was only suppressed at high H_2_O_2_ doses (Figure 2B). RUNX2 expression was reduced independently of changes in mRNA expression, whereas the reduction in OSX protein expression was preceded by decreased *Sp7* mRNA expression (Supplemental Figure 3, C and D). These data suggest that H_2_O_2_ directly suppresses RUNX2 protein expression and secondarily reduces OSX expression downstream of changes in mRNA. Increasing ROS by inhibiting GCLC similarly reduced RUNX2 protein in naive calvarial cells without affecting mRNA expression (Supplemental Figure 3, E and F). To determine if RUNX2 downregulation was a direct or indirect effect due to altered osteoblast potential, we expressed Flag-tagged RUNX2 in HEK293 cells, a nonosteogenic cell line. H_2_O_2_ treatment induced PRDX1-SO_3_ and reduced exogenous RUNX2 expression in a dose-dependent manner (Supplemental Figure 3G). GCLC inhibition also significantly reduced exogenous RUNX2 with almost a complete loss after 48 hours (Supplemental Figure 3H). Moreover, RUNX2 downregulation occurred rapidly in naive calvarial cells, within 45 minutes after addition of H_2_O_2_ (Supplemental Figure 3I). Based on these data, we hypothesized that RUNX2 is oxidized and degraded in response to ROS. To evaluate protein oxidation, we utilized a probe with the sulfenic acid–reactive 3-(2,4-dioxocyclohexyl) propyl (DCP) linked to biotin (DCP-Bio1) to characterize cellular oxidation of cysteine residues (Supplemental Figure 3J). H_2_O_2_ treatment and GCLC inhibition in calvarial cells increased DCP-Bio incorporation into total protein, indicative of large-scale protein oxidation (Supplemental Figure 3, K and L). DCP-Bio was enriched in both endogenous RUNX2 in naive calvarial cells and Flag-tagged RUNX2 in HEK293 cells following H_2_O_2_ treatment (Figure 2C and Supplemental Figure 3M). These results indicate that RUNX2 is oxidized on cysteine residues in the presence of elevated ROS. Cysteine oxidation can cause conformational changes of proteins and ultimately lead to degradation via the 20S proteasome (52, 53). Cycloheximide (CHX) chase experiments demonstrated that increasing ROS reduced the half-life of RUNX2 from 8 hours to 2 hours (Figure 2, D and E). This was attributed to increased proteasomal degradation, as MG132 prevented RUNX2 downregulation in response to H_2_O_2_ (Figure 2F). These data indicate that, when GSH is limited, RUNX2 is directly oxidized by ROS and subsequently degraded. Consistent with this, supplementing cell-permeable GSH mono-ethyl ester (MEE-GSH) prevented ROS accumulation and protein oxidation and rescued RUNX2 expression in naive calvarial cells treated with BSO (Supplemental Figure 4, A and B). Importantly, increasing RUNX2 expression erased the effects of H_2_O_2_ at lower concentrations and enhanced osteoblast differentiation (Figure 2, G and H, and Supplemental Figure 4C). However, exogenous RUNX2 was unable to rescue osteoblast differentiation at higher H_2_O_2_ concentrations, likely due to degradation of both the endogenous and FLAG-RUNX2 (Figure 2, G and H, and Supplemental Figure 4C). Collectively, these data indicate that ROS inhibits osteoblast differentiation primarily through the oxidation and degradation of RUNX2.

### Maintaining GSH is the essential function of glutamine metabolism in osteoblasts.

**Figure 3:**
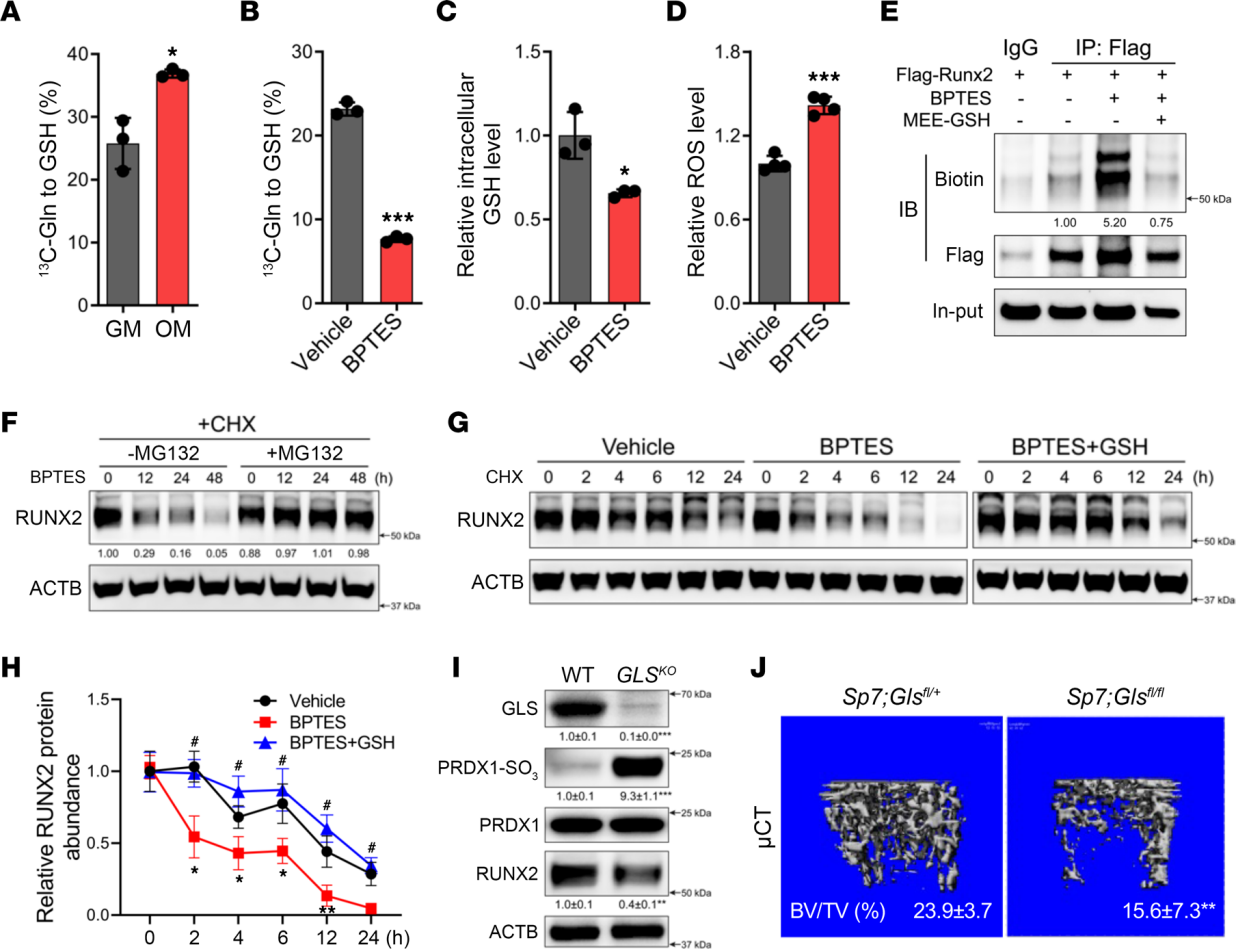
Glutamine metabolism is essential for maintaining GSH concentration to stabilize RUNX2. (**A**) Fractional contribution of [U-^13^C]glutamine to GSH in naive and differentiated calvarial cells (*n* = 3). (**B**–**D**) Effect of GLS inhibition using BPTES on the contribution of [U-^13^C]glutamine to GSH (**B**), intracellular GSH levels (**C**), and ROS levels (**D**) in calvarial cells (*n* = 3-4). (**E**) Effect of BPTES and MEE-GSH treatment on biotinylated-dimedone incorporation into immunoprecipitated FLAG-RUNX2 in HEK293 cells (*n* = 3). (**F**) Western blot analysis of the effect of MG132 on RUNX2 expression in calvarial cells treated with BPTES for up to 48 hours (*n* = 3). (**G** and **H**) Western blot analysis of the effect of BPTES and MEE-GSH on RUNX2 degradation (*n* = 3). Quantification of 3 independent experiments is shown in **H**. (**I**) Western blot analysis of GLS, PRDX1-SO_3_, PRDX1, and RUNX2 in bone extracts isolated from *Sp7Cre;Gls^fl/+^* (WT) and *Sp7Cre;Gls^fl/fl^* (*GLS^KO^*) mice (*n* = 4). PRDX1-SO_3_ was normalized to total PRDX1; all other proteins were normalized to ACTB. (**J**) Representative μCT images of distal femurs from WT and *GLS^KO^* mice (*n* = 11). BV/TV is listed below each image. Data are shown as mean ± SD. *P < 0.05, **P < 0.01, ***P < 0.001. ^#^*P* < 0.05, comparison between group BPTES and BPTES+GSH. Two-tailed Student’s unpaired *t* test (**A**–**D** and **H**) and 2-tailed Student’s paired *t* test (**I** and **J**) were used.

Our data indicate that GSH is essential in the regulation of RUNX2 stability by neutralizing ROS. We next sought to test these conclusions by modulating GSH concentrations independently of changes in GCLC expression or activity. Glutamine metabolism contributes to GSH biosynthesis by providing glutamate downstream of the enzyme GLS (Supplemental Figure 5A). Indeed, stable isotopomer analyses found that [U-^13^C]glutamine contribution to GSH increased significantly during differentiation (Figure 3A). Next, we targeted glutamine metabolism by either limiting glutamine availability or inhibiting GLS pharmacologically, using Bis-2-(5-phenylacetamido-1,3,4-thiadiazol-2-yl)ethyl sulfide (BPTES), or genetically, using CRISPR/Cas9 targeting, and we evaluated the effects on GSH and oxidative stress (Supplemental Figure 5A). These approaches all resulted in reduced flux of glutamine carbon into GSH, decreased intracellular GSH concentration, increased ROS accumulation, and impaired osteoblast differentiation (Figure 3, B–D, and Supplemental Figure 5, B–F). Importantly, GLS inhibition increased both general protein and RUNX2 oxidation and reduced RUNX2 half-life due to proteasomal degradation (Figure 3, E–H, and Supplemental Figure 5, G and H). Addition of MEE-GSH completely negated the effects of GLS inhibition and reduced ROS, prevented RUNX2 oxidation, and rescued RUNX2 stability (Figure 3, E, G, and H, and Supplemental Figure 5, I and J).

To corroborate these findings in vivo, we deleted a floxed allele of *Gls* (*Gls^fl^*) using *Sp7Cre* (Figure 3I). As expected, primary bone cells migrated from cultured *Sp7Cre;Gls^fl/fl^* bone shaft exhibited reduced GLS activity (Supplemental Figure 5K). μCT analysis found that BV/TV, Tb.N, Tb.Th, and BMD were significantly reduced, while Tb.Sp was significantly increased in both male and female *Sp7Cre;Gls^fl/fl^* animals at 4 months of age (Figure 3J and Supplemental Table 2). Cortical bone was also affected in *Sp7Cre;Gls^fl/fl^* animals, with reductions in both Ct.Th and T.Ar (Supplemental Table 2). *Sp7Cre;Gls^fl/fl^* mice had significantly fewer OCN^+^ osteoblasts per bone with no difference in the number of TRAP^+^ osteoclasts per bone compared with *Sp7Cre;Gls^fl/+^* littermate controls (Supplemental Table 2). *Gls* ablation also impaired osteoblast activity, as both the MAR and BFR were significantly reduced in the *Sp7Cre;Gls^fl/fl^* mice compared with *Sp7Cre;Gls^fl/+^* littermates (Supplemental Table 2). Consistent with the in vitro findings, primary bone cells migrated from cultured *Sp7Cre;Gls^fl/fl^* bone shaft had reduced contribution of glutamine carbon into GSH and increased ROS (Supplemental Figure 5, L and M). Moreover, *Sp7Cre;Gls^fl/fl^* bones had increased PRDX1-SO_3_ and significantly less RUNX2 protein (Figure 3I). Thus, maintaining intracellular GSH may be the primary function of glutamine metabolism in osteoblasts.

**Figure 4:**
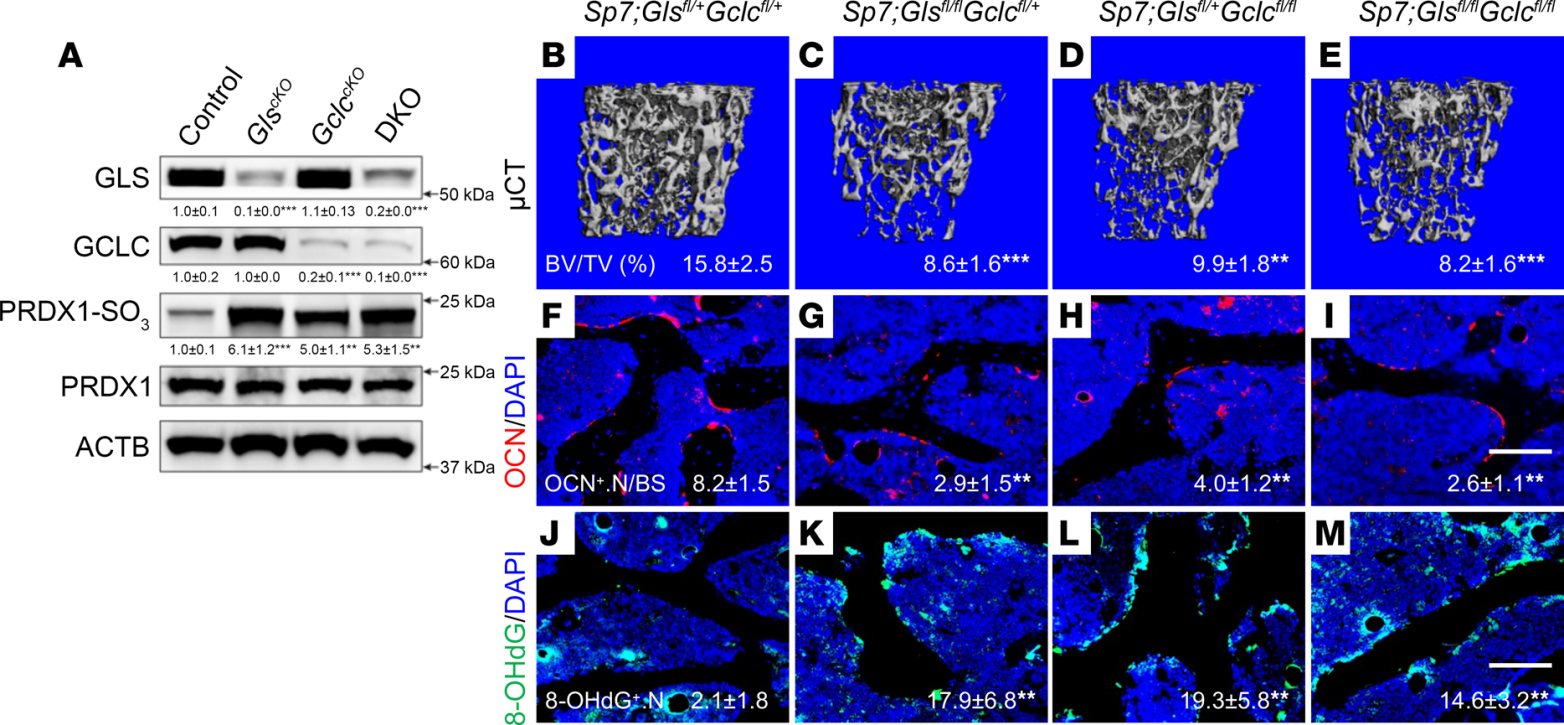
*Gls* and *Gclc* function in the same molecular pathway in osteoblasts. (**A**) Western blot analysis of GLS, GCLC, PRDX1-SO_3_, and PRDX1 in bone extracts (*n* = 4). PRDX1-SO_3_ was normalized to total PRDX1; all other proteins were normalized to ACTB. (**B**–**E**) Representative μCT images of trabecular bone from distal femur of 4-month-old mice (*n* = 6–10 per genotype). BV/TV is listed below each image. (**F**–**M**) Representative histological sections showing osteocalcin (OCN) immunofluorescence (**F**–**I**) or 8-OHdG immunofluorescence (**J**–**M**) performed in distal femurs from *Sp7Cre;Gls^fl/+^Gclc^fl/+^* (control), *Sp7Cre;Gls^fl/fl^Gclc^fl/+^* (*GLS^cKO^*), *Sp7Cre;Gls^fl/+^Gclc^fl/fl^* (*GCLC^cKO^*), and *Sp7Cre;Gls^fl/fl^Gclc^fl/fl^* (DKO) mice (*n* = 6–10). Scale bar: 100 μm. Quantifications of the individual stains are listed below each image. Data are shown as mean ± SD. **P < 0.01, ***P < 0.001 (all groups were compared with control group). Two-way ANOVA was used.

To further investigate this link, we generated mice lacking either *Gls*, *Gclc*, or both in osteoblasts using *Sp7Cre*. This strategy effectively ablated GLS and/or GCLC protein from bone tissue of the respective mutant mice (Figure 4A). Conditional ablation of either GLS or GCLC resulted in a similar statistically significant reduction of bone mass (Figure 4, B–D, and Supplemental Table 3). Interestingly, bone mass was not further reduced in *Sp7Cre;Gls^fl/fl^Gclc^fl/fl^*–double KO (*Sp7Cre;Gls^fl/fl^Gclc^fl/fl^*-DKO) mice as compared with either the *Gls*-mutant (*Sp7Cre;Gls^fl/fl^Gclc^fl/+^)* or *Gclc-*mutant (*Sp7Cre;Gls^fl/+^Gclc^fl/fl^*) mice (Figure 4, B–E, and Supplemental Table 3). Likewise, static histomorphometry found a similar reduction in overall osteoblast numbers in both the *Gls*-mutant and *Gclc*-mutant mice, with no additive or synergetic effect observed in the DKO mice (Figure 4, F–I, and Supplemental Table 3). Serum analysis found a significant reduction of both P1NP and OCN in both the *Gls*-mutant and *Gclc*-mutant mice, with no change in C-terminal telopeptides of type I collagen (CTX-I) (Supplemental Table 3). Interestingly, the DKO mice had lower serum P1NP compared with either *Gls*-mutant or *Gclc*-mutant mice but had no further reduction of serum OCN (Supplemental Table 3). Both *Gls*- and *Gclc*-mutant bones were characterized by increased protein and DNA oxidation, as determined by PRDX1-SO_3_ and 8-OHdG immunostaining, respectively (Figure 4, A and J–L). Interestingly, the levels of protein and DNA oxidation were not significantly different in the DKO mice (Figure 4, A and M). Despite elevated protein and DNA oxidation, we did not observe TUNEL^+^ osteoblasts under any of these conditions (Supplemental Figure 6). Collectively, these data indicate that GLS and GCLC function in the same molecular pathway to regulate GSH biosynthesis necessary for osteoblast differentiation and bone formation in mice.

### Reducing ROS stabilizes RUNX2 when GSH is limited.

**Figure 5:**
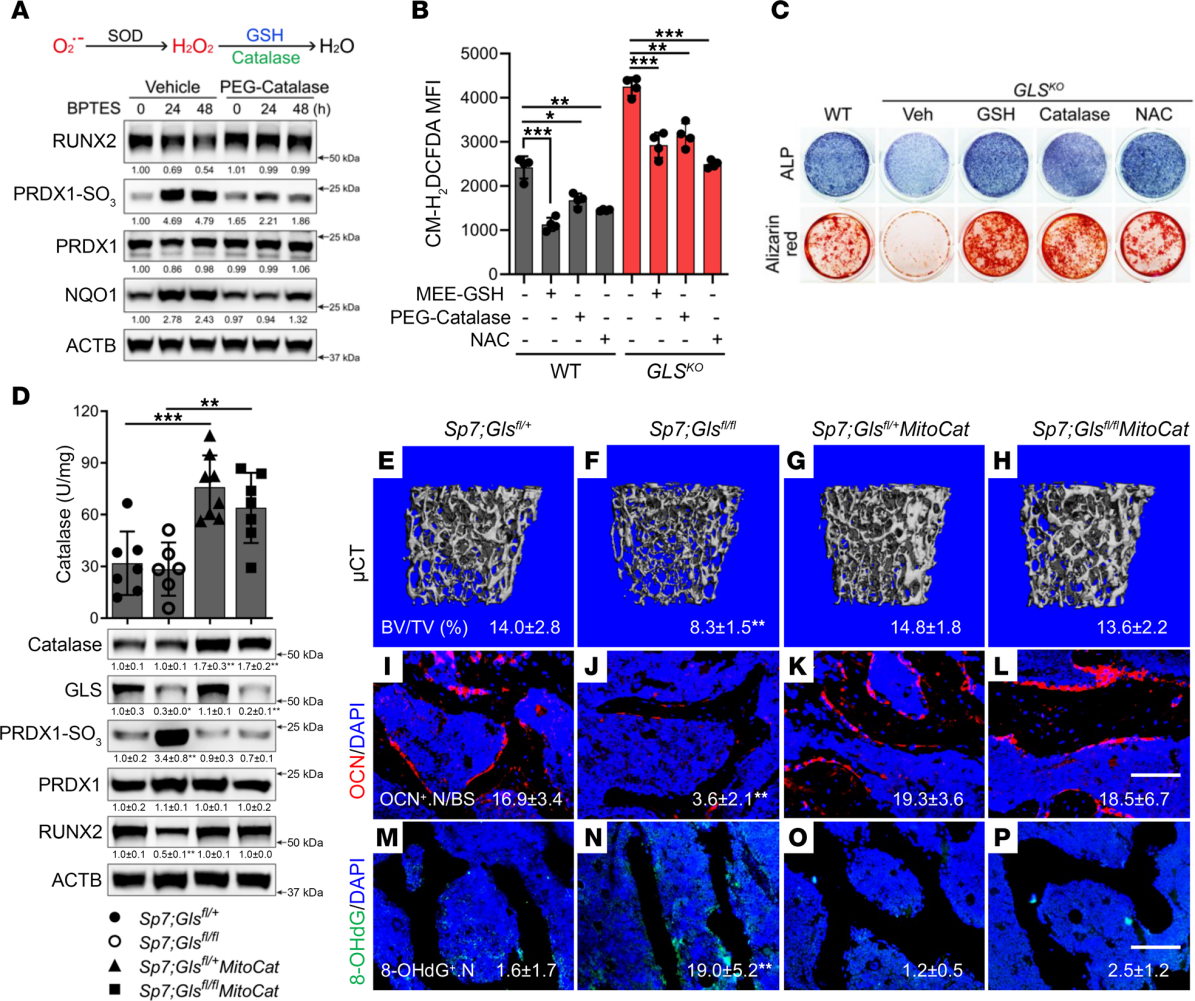
Eliminating ROS stabilizes RUNX2 and restores osteoblast differentiation and bone formation when GSH is limited. (**A**) Schematic of catalase function and Western blot analysis of the effect of PEG-CAT on protein expression in calvarial cells treated with BPTES for the indicated time courses (*n* = 3). (**B** and **C**) Evaluation of the effects of various antioxidants on ROS levels (**B**) or osteoblast differentiation (**C**) in calvarial cells isolated from *Sp7Cre;Gls^fl/+^* (WT) or *Sp7Cre;Gls^fl/fl^* (*GLS^KO^*) mice (*n* = 3–4). (**D**) Analyses of catalase activity and protein expression in bone extracts from *Sp7Cre;Gls^fl/+^*, *Sp7Cre;Gls^fl/fl^*, *Sp7Cre;Gls^fl/+^MitoCat*, and *Sp7Cre;Gls^fl/fl^MitoCat* mice (*n* = 6–8). PRDX1-SO_3_ was normalized to total PRDX1; all other proteins were normalized to ACTB. (**E**–**P**) Representative μCT images (**E**–**H**), OCN (**I**–**L**), or 8-OHdG immunostaining (**M**–**P**) of distal femurs from *Sp7Cre;Gls^fl/+^*, *Sp7Cre;Gls^fl/fl^*, *Sp7Cre;Gls^fl/+^MitoCat*, and *Sp7Cre;Gls^fl/fl^MitoCat* mice (*n* = 6–10). All groups were compared with control (*Sp7Cre;Gls^fl/+^*) group. Scale bar: 100 μm. BV/TV and quantifications are listed below each image. Data are shown as mean ± SD. *P < 0.05, **P < 0.01, ***P < 0.001. Two-way ANOVA (**B**, **D**, and **E**–**P**) was used.

We next sought to determine whether reducing ROS can rescue the impaired osteoblast differentiation and bone formation when GSH is limited. We treated naive calvarial cells with pegylated CAT (PEG-CAT), an enzyme that neutralizes H_2_O_2_ independently of GSH (Figure 5A). Like MEE-GSH, PEG-CAT treatment reduced PRDX1-SO_3_ and NQO1 induction and prevented RUNX2 degradation caused by GLS inhibition (Figure 5A). Next, we evaluated the effect of several different antioxidants on ROS and osteoblast differentiation in primary *Sp7Cre;Gls^fl/fl^* calvarial cells. Supplementation with either MEE-GSH, PEG-CAT, or N-acetylcysteine (NAC) reduced ROS and rescued osteoblast differentiation when glutamine metabolism was inhibited (Figure 5, B and C, and Supplemental Figure 7). However, addition of other metabolites (such as glutamate or α-KG), which were unable to neutralize ROS or limit oxidative stress, were unable to rescue osteoblast differentiation (Supplemental Figure 7, C–E). To test these results in vivo, we generated *Sp7Cre;Gls^fl/fl^* mice that also expressed a human CAT transgene targeted to the mitochondria (*Sp7Cre;Gls^fl/fl^;MitoCat*). Expression of the *MitoCat* transgene resulted in a 2-fold increase of CAT protein expression and CAT activity in bone tissues from *Sp7Cre;MitoCat* transgenic mice as compared with their littermate control (Figure 5D). PRDX1-SO_3_ and 8-OHdG were significantly reduced in bone tissue from *Sp7Cre;Gls^fl/fl^;MitoCat* mice when compared with *Sp7Cre;Gls^fl/fl^* littermates (Figure 5, D and M–P). Moreover, RUNX2 expression was restored in *Sp7Cre;Gls^fl/fl^;MitoCat* mice compared with *Sp7Cre;Gls^fl/fl^* littermates (Figure 5D). Consistent with this, bone mass as well as other bone parameters (Tb.N, Tb.Th, and Tb.Sp) were rescued to WT levels in *Sp7Cre;Gls^fl/fl^;MitoCat* mice when compared with *Sp7Cre;Gls^fl/fl^* mice (Figure 5, E–H, and Supplemental Table 4). In fact, *Sp7Cre;Gls^fl/fl^;MitoCat* mice were indistinguishable from *Sp7Cre;Gls^fl/+^* WT controls (Figure 5, E–H, and Supplemental Table 4). Moreover, MitoCAT expression rescued both the number of OCN^+^ osteoblasts per bone surface and osteoblast activity caused by *Gls* deletion, as indicated by static and dynamic histomorphometry and serum P1NP and OCN (Figure 5, I–L, and Supplemental Table 4). Collectively, these data highlight the essential role for glutamine-dependent GSH biosynthesis to limit ROS and stabilize RUNX2 to promote osteoblast differentiation and bone formation.

### Reducing ROS improves bone development in the Runx2^+/–^ haplo-insufficient mouse model.

**Figure 6:**
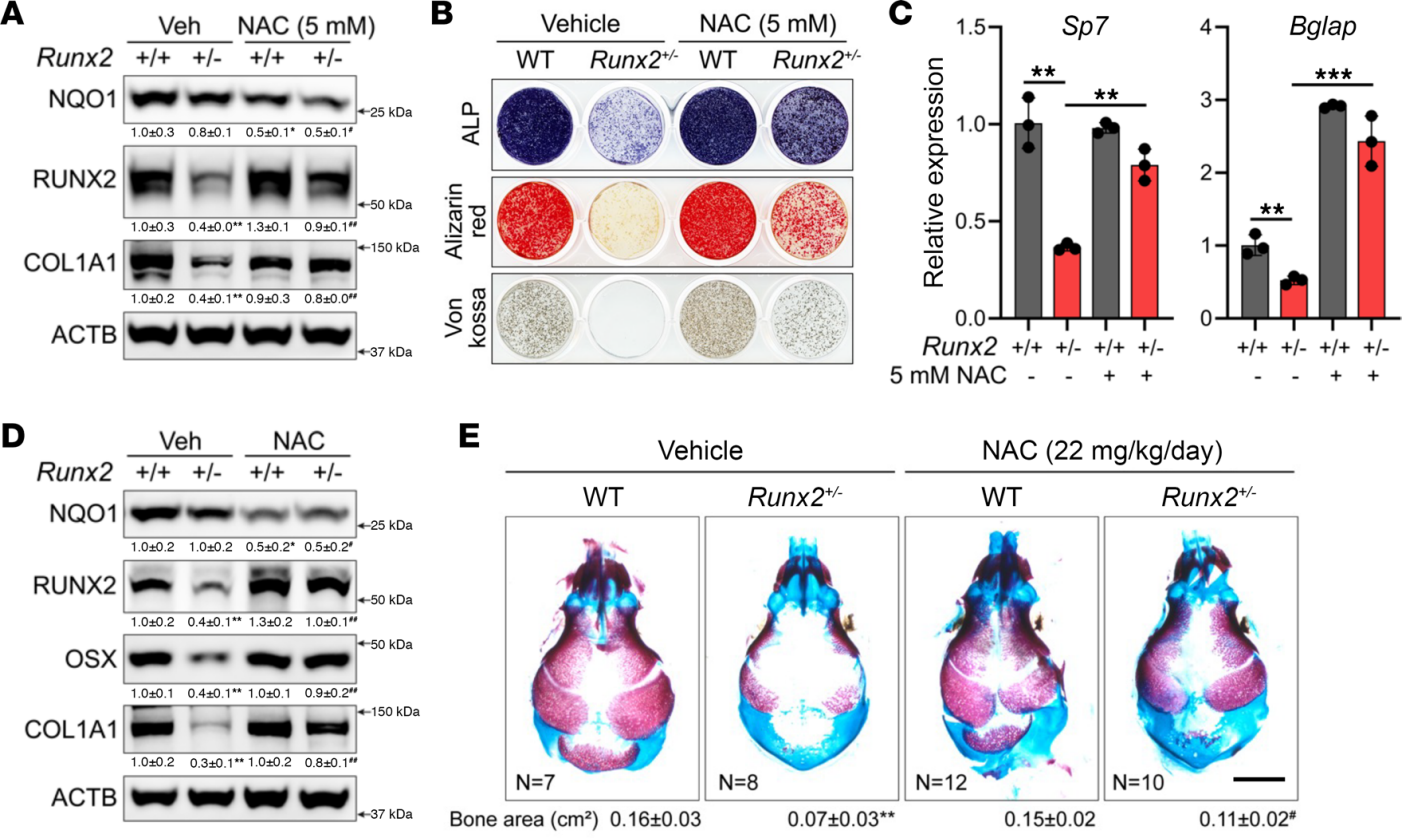
NAC restores RUNX2 expression and improves bone development in *Runx2^+/−^* embryos. (**A**–**C**) Effect of NAC on protein expression (**A**), functional assays of osteoblast differentiation (**B**), or gene expression determined by quantitative PCR (qPCR) (**C**) in calvarial cells isolated from WT and *Runx2^+/−^* mice (*n* = 3). (**D**) Western blot analysis of NQO1, RUNX2, OSX, and COL1A1 in the skull of E18.5 WT and *Runx2^+/−^* embryos carried by NAC- or vehicle-treated mothers (*n* = 3). (**E**) Alcian blue/alizarin red–stained skeletal preparations and quantification of the mineralized area of the skull of E18.5 *Runx2^+/−^* embryos carried by NAC- or vehicle-treated mothers (*n* = 7–12). Scale bar: 2 mm. All proteins were normalized to ACTB. Data are shown as mean ± SD. **P < 0.01, ***P < 0.001. * denotes comparison with control (vehicle-treated *Runx2^+/+^*) group; # denotes comparison between vehicle-treated *Runx2^+/–^* and NAC-treated *Runx2^+/–^* groups. Two-way ANOVA (**A** and **C**–**E**) was used.

Our data indicate that, when ROS was elevated, RUNX2 was rapidly oxidized and degraded. However, whether this is a physiological or pathological phenomenon is not known. To test this, we used the *Runx2^+/–^* haplo-insufficient mouse, which has reduced RUNX2 expression and CCD. Calvarial cells isolated from *Runx2^+/–^* mice have an approximately 60% reduction in RUNX2 protein as well as reduced expression of COL1A1, a downstream target of RUNX2 (Figure 6A). It is important to note that loss of 1 allele of *Runx2* did not increase ROS, as determined by NQO1 expression (Figure 6A). However, treatment of *Runx2^+/–^* calvarial cells with the antioxidant NAC reduced NQO1 expression, increased RUNX2, and rescued COL1A1 expression (Figure 6A). Consistent with increased RUNX2 expression, NAC improved osteoblast differentiation in vitro, as evidenced by increased expression of *Sp7* and *Bglap* and increased matrix mineralization (Figure 6, B and C). We next determined whether NAC could improve RUNX2 expression and bone formation in *Runx2**^+/–^*** embryos. To this end, pregnant dams were provided water containing 1 mM NAC ad libitum from conception until E18.5. We focused our analysis on calvarial bones that form by intramembranous ossification, as they are the ones affected by *Runx2* haplo-insufficiency. *Runx2^+/–^* mice did not have increased NQO1 expression in parietal bone protein extracts, suggesting that they did not experience increased ROS (Figure 6D). NAC treatment reduced NQO1 expression in both *Runx2^+/+^* (WT) and *Runx2^+/–^* littermates, indicative of decreased ROS in calvarial bones (Figure 6D). Consistent with reduced ROS, NAC restored RUNX2 expression and rescued osteoblast differentiation, as determined by OSX and COL1A1 expression (Figure 6D). Alcian blue/Alizarin red staining showed that the mineralized area in interparietal bones was increased by 1.5-fold in *Runx2**^+/–^*** embryos carried by NAC-treated mothers compared with those carried by vehicle-treated mothers (Figure 6E). No effect of NAC treatment was observed in either WT or *Runx2^–/–^* embryos carried by NAC-treated mothers, although NAC treatment did increase RUNX2 expression in WT embryos (Figure 6E and Supplemental Figure 8A). This indicates that RUNX2 is required for the beneficial effects of NAC on bone development in this model. In contrast to the calvarium, we did not observe any difference in sternum or clavicle development in *Runx2**^+/–^*** embryos carried by NAC-treated mothers (Supplemental Figure 8B).

## Discussion

The critical transcriptional regulations governing osteoblast differentiation have been extensively investigated, while little is known about the metabolic rewiring in differentiating osteoblasts. Similar to the Warburg effect in cancer cells, differentiating osteoblasts also exhibit a unique metabolic feature favoring aerobic glycolysis, whereas the naive osteoblast progenitors mainly rely on OXPHOS (16). The metabolic reprogramming to fulfill the increased energetic/biosynthetic and other demands in differentiating osteoblasts provides a hint that metabolic manipulation might be a strategy to induce bone formation. In the current study, we have unveiled a link between GSH biosynthesis and bone mass, specifically through the neutralization of ROS that oxidize and degrade RUNX2 and prevent osteoblast differentiation and bone formation. When GSH biosynthesis is limited, ROS increase and oxidize cysteine residues in RUNX2. Oxidized RUNX2 undergoes proteasomal degradation, and osteoblast differentiation is subsequently inhibited. Reducing ROS even when GSH biosynthesis is limited stabilizes RUNX2 and facilitates osteoblast differentiation and bone formation. These data are consistent with recent data demonstrating that GSH levels increase during osteoblast differentiation and that ROS must be tightly controlled for osteoblast differentiation to proceed (14, 33, 54). Thus, GSH biosynthesis is essential for osteoblast differentiation by establishing a permissive REDOX environment.

As in other tissues, excessive ROS is known to have deleterious effects to the skeleton and are associated with the loss of bone mass caused by skeletal aging or sex steroid deficiency (30–32). The negative effects of ROS on bone are well known but have been historically linked primarily to osteoclasts. ROS accumulation prolongs the survival of osteoclast precursors and is essential for RANKL-induced osteoclast differentiation and bone resorption (55, 56). By comparison, ROS is detrimental in osteoblasts. In osteoblast progenitor cells, ROS attenuates canonical Wnt/β-catenin–induced osteogenesis by diverting β-catenin away from T cell factor/lymphoid enhancer factor (TCF/LEF) to FoxO-mediated transcription of antioxidant genes (57, 58). Moreover, oxidized lipids can bind to and activate PPARγ to favor adipogenesis and increase BM adiposity at the expense of osteogenesis (59). In addition to these mechanisms, we posit that ROS directly regulates RUNX2 stability to influence osteogenic differentiation. Several studies have linked GSH and/or ROS to RUNX2 in different contexts. For example, ROS inhibited osteoblast differentiation in MC3T3 cells by reducing RUNX2 expression and activity either through transcriptional repression or by affecting the transactivation of downstream target genes downstream of NRF2 (60, 61). In contrast to these studies, our data indicate that ROS reduces RUNX2 protein expression independently of changes in mRNA expression. Other studies found that BSO-induced bone loss is mediated by TNF-α, which can regulate RUNX2 degradation downstream of SMURF1 (62, 63). While we observed rapid degradation of RUNX2, and we did not observe increased TNF-α expression in our models (data not shown). Rather, our data suggest that RUNX2 is directly oxidized by ROS. In response to multiple methods of increasing ROS, we observed cysteine oxidation and subsequent degradation of RUNX2. Consistent with this, reducing ROS in these conditions using antioxidants protected and stabilized RUNX2 and promoted osteoblast differentiation. Consistent with a recent study, we observed that reducing endogenous ROS in WT cells by expressing CAT did not promote osteoblast differentiation or bone formation in otherwise WT mice (64). Thus, we conclude that ROS is normally maintained below a threshold that must be reached before RUNX2 is oxidized and degraded. If ROS rises above this threshold, RUNX2 is rapidly oxidized and degraded and osteoblast differentiation is inhibited. In conditions of elevated ROS, RUNX2 degradation may provide some benefit to the osteoblast by limiting mitochondrial dysfunction. Elevated ROS inhibit mitochondrial function by feeding a “vicious circle” involving mitochondrial DNA damage, decreased expression of respiratory chain proteins, further ROS generation, and dysfunctional mitochondrial stress response (65, 66). Rapid degradation of RUNX2 should limit glucose consumption and reduce OXPHOS, reducing ROS levels and sparing the mitochondria. This likely explains the lack of senescence or apoptotic markers in GSH-deficient osteoblasts in vivo. Collectively, these data indicate that osteoblast differentiation relies on a well-controlled REDOX environment and RUNX2 functions as a molecular sensor to coordinate osteoblast differentiation with ROS. If the conditions for osteoblast differentiation are not optimal (e.g., elevated ROS), RUNX2 is oxidized and degraded — effects that limit osteoblast differentiation until a permissive REDOX environment is established.

The primary source of ROS in osteoblasts is not entirely clear. Differentiating osteoblasts are characterized by robust mitochondrial biogenesis and increased oxygen consumption ostensibly to fulfill the energy demand associated with increased protein synthesis (12, 13, 15). While the precise contribution of either OXPHOS or protein synthesis to ROS levels was not investigated, both processes likely contribute to ROS generation in osteoblasts. Our data suggest that mitochondrial ROS is the most likely culprit, as expressing the mitochondrial targeted CAT transgene rescued both osteoblast and bone parameters under GSH-depleted conditions (Figure 5). Nevertheless, considering the high rates of protein synthesis in differentiating osteoblasts, oxidative protein folding in the ER likely also contributes to ROS accumulation. Thus, the distribution of ROS in different subcellular compartments needs further evaluation.

Another important takeaway from our study is the essential nature of glutamine metabolism to establish a permissive REDOX environment in osteoblasts. In cancer cells, no less than 30% of imported glutamine was shown to leave the cell through the xCT transporter as glutamate in exchange for cysteine, the rate-limiting precursor for GSH biosynthesis (67). Thus, the availability of 2 of the 3 constituent amino acids of GSH relies on GLS-mediated glutamine metabolism. Osteoblast-specific ablation of GLS phenocopied the effects of GCLC ablation, namely decreased GSH levels, increased ROS, reduced RUNX2, and reduced bone formation. Previous studies, including our own, have attributed many of the cellular effects of GLS KO to different metabolites; glutamine-derived α-KG regulates proliferation in skeletal stem cells, and glutamine-derived amino acids promote protein and bone matrix synthesis in pre-osteoblasts (19, 20, 46, 47). In contrast, GSH appears to be the essential product of glutamine metabolism in *Sp7-*expressing osteoblasts. For example, GLS ablation did not enhance the GCLC-KO bone phenotype nor result in additional bone phenotypes, suggesting these 2 enzymes function in the same molecular pathway. Consistent with this, supplementing GLS-deficient cells with exogenous GSH rescued differentiation and matrix mineralization in vitro, whereas other metabolites (e.g., glutamate, α-KG) could not. Our results differ from a recent study, which found that GSH supplementation was unable to rescue osteoblast differentiation in GLS-deficient cells (46). Our study differed from this paper in 2 important variables: the concentration of GSH used for the rescue and the source of osteoblasts. Here, we used 2.5 mM GSH compared with 1 mM reported in Stegen et al. (46). We used 2.5 mM to more accurately replenish intracellular GSH, which is reported to be between 2 and 5 mM in mammalian cells (68). Consistent with this, 2.5 mM GSH could reduce intracellular ROS back to WT levels in *Gls-*deficient cells (Supplemental Figure 5I). A second difference was that Stegen et al. used FACS to sort Sp7^+^ cells from the long bone, whereas we used primary calvarial cells isolated from newborn mice. We cannot rule out that osteoblasts from different developmental origins have unique metabolic requirements or use glutamine differently. However, in support of the primacy of glutamine-derived GSH, supplementing GSH but not other nutrients (e.g., glutamate or α-KG) rescued osteoblast differentiation in the absence of glutamine. Furthermore, reducing ROS (independently of GSH) using exogenous CAT or by CAT overexpression increased RUNX2 and rescued osteoblast differentiation and bone formation in GLS-deficient mice (Figure 5). Thus, we conclude that SP7^+^ osteoblasts in long bones can tolerate the reduction in glutamine-derived αKG and amino acids if ROS and oxidative stress are controlled. Highlighting the specificity of increased ROS to the GLS-KO bone phenotype, CAT overexpression could not rescue bone mass in ATG7-deficient osteoblasts despite reducing ROS burden (64). Taken together, we conclude that maintaining intracellular GSH is the primary function of glutamine metabolism in differentiating osteoblasts. Moreover, our data support the notion that metabolic manipulations such as stimulating glutamine metabolism or cysteine uptake are viable strategies to increase RUNX2 and boost bone formation downstream of increased GSH biosynthesis. Thus, dietary manipulation of glutamine metabolism and GSH biosynthesis may provide a benefit not only in CCD patients but also patients with low bone mass due to aging or the loss of sex steroids. Future studies into this area are warranted.

In the rescue experiments, it was peculiar that glutamate supplementation could only rescue alkaline phosphatase expression but could not rescue osteoblast differentiation or oxidative stress, even though glutamate is essential for GSH synthesis. We think this is likely due to a combination of events. First, a recent study found that glutamate supplementation increased ALP expression by activating PKC and Erk1/2 downstream of the glutamate receptor (GluR) (69). Second, a separate study found that glutamate supplementation reduced intracellular GSH by inhibiting cysteine uptake in C3H10T1/2 cells. Moreover, glutamate supplementation in C3H10T1/2 cells increased ROS, reduced nuclear RUNX2 expression and activity, and inhibited osteoblast differentiation (70). Based on these studies, we conclude that increasing extracellular glutamate inhibits cysteine uptake to limit GSH synthesis and activates GluR to increase ALP expression.

Maintaining GSH biosynthesis has been shown to be essential in neutralizing ROS and regulating osteoblast and chondrocyte viability (46, 71, 72). Similarly, in the settings of aging or estrogen deficiency, accumulated ROS promotes osteoblast/osteocyte apoptosis mainly through a PKC-β/p66shc/NF-κB signaling cascade (31). Interestingly, we did not observe reduced viability or increased cell apoptosis/senescence in any model where we inhibited GSH biosynthesis either genetically or pharmacologically. This may be because of compensatory upregulation of other antioxidant systems like CAT or thioredoxins that can reduce H_2_O_2_ independently of GSH. Consistent with this, we observed increased expression of numerous antioxidant genes and proteins in both the GCLC- and GLS-deficient cells. This likely maintains ROS at subtoxic levels that cause oxidative damage (e.g., DNA and protein oxidation) and inhibit osteoblast differentiation without causing cell death. This is akin to a flat tire on a car; the car remains functional but cannot reach its destination unless the tire is fixed. Here, the osteoblast progenitor remains viable but is unable to further differentiate due to the elevated ROS burden and RUNX2 degradation. When the ROS burden is lowered, using exogenous antioxidants or expressing a mitochondrial targeted CAT, the cell can resume the differentiation process due to RUNX2 stabilization.

## Methods

Please find the manufacturer information for all products and antibodies in Supplemental Table 7 (Key Resources Table).

### Mouse strains.

*Gls^fl^* and *Sp7Cre* mouse strains are as previously described. *C57Bl/6 J*, *Rosa26^Cas9^*, *Rosa26^Flpe^*, and *MitoCat* mouse strains were obtained from the Jackson Laboratory. *Gclc^lacZ^* (C57BL/6NTac-Gclc^tm1a(EUCOMM)Wtsi^/WtsiCnbc) was purchased from the European Mouse Mutant Archive (www.infrafrontier.eu/emma/). *Gclc^fl^* allele was generated by crossing *Gclc^lacZ^* to *Rosa26^Flpe^* mouse strains to remove the FRT-flanked *LacZ* cassette, followed by backcrossing with C57BL/6J to remove the *Rosa26^Flpe^* allele. The *Runx2^null^* allele was generated as previously described (3, 8) and was a gift from Gerard Karsenty (Columbia University, New York City, New York, USA). The *Sp7Cre* mice have a partially penetrant bone phenotype. To control for this, in all genetic experiments, *Sp7Cre;Gclc^fl/fl^*-KO mice are always compared with *Sp7Cre;Gclc^fl/+^* as WT littermate controls. All mice were housed at 23°C on a 12-hour light/dark cycle with free access to water and PicoLab Rodent Diet 290. For NAC administration, mice were fed with water supplemented with 1 mM NAC. NAC as a 0.5 M stock was prepared in ddH_2_O once a month, aliquoted, and stored in the dark at −20°C. Sterile drinking water was freshly made from the stock and changed every 3 days. For all animal studies, both male and female mice were analyzed. The assessment of all animals was performed in a blinded and coded manner.

### Primary cells and cell lines.

Primary murine calvarial cells were isolated from newborn mice (P3–P5) and cultured in α-MEM containing 15% FBS. The HEK293 cell line was purchased from ATCC and cultured in low-glucose DMEM containing 10% FBS. All cell culture experiments were carried out at 37°C and 5% CO_2_.

### Mouse analysis.

μCT (VivaCT 80, Scanco Medical AG, set at 55 kVp and 145 μA) was used for 3-dimensional reconstruction and quantification of bone parameters. For quantification of bone mass in the long bone, femurs were isolated, fixed, immobilized, and scanned. Bone parameters were quantified from 200 slices directly underneath the growth plate with the threshold set at 320.

### Histomorphometry and immunostaining.

Bone static histomorphometry was performed on femurs fixed in 10% buffered formalin for 48 hours at 4°C followed by 2-week decalcification in 14% EDTA. Decalcified bones were embedded in paraffin and sectioned at 5 μm thickness. H&E and tartrate-resistant acid phosphatase (TRAP) staining were performed following standard protocols. For dynamic histomorphometry, alizarin red (60 mg/kg) and calcein (20 mg/kg) were i.p. injected at 7 and 2 days prior to sacrifice, respectively. Freshly isolated bones were fixed in 4% paraformaldehyde overnight and dehydrated in 30% sucrose for 24 hours and embedded in OCT for frozen sections using CryoJane Tape-Transfer System (Electron Microscopy Sciences). Both static and dynamic histomorphometry were quantified using ImageJ. For immunostaining, conditions were slightly adapted according to the type of antibody used. For OCN immunostaining, antigen retrieval was performed by incubating tissue sections in 10 μg/mL proteinase K for 10 minutes, followed by incubation in 3% H_2_O_2_ (v/v in Methanol) for 10 minutes to block endogenous peroxidase activity. For 8-OHdG staining, antigen retrieval was performed by boiling the slides in citric acid–based antigen unmasking solution for 10 minutes. Subsequently, sections were incubated overnight at 4°C with primary antibody against OCN or 8-OHdG. TUNEL staining was performed with an In Situ Cell Death Detection Kit. Sections were permeabilized for 2 minutes on ice in 0.1% sodium citrate containing 0.1% Triton X-100 and then incubated with TUNEL reaction mixture for 1 hour at 37°C. All image analysis and quantifications was performed in a blinded and coded manner.

### Skeletal preparations.

Timed pregnant females were euthanized, and pups were harvested at E18.5. The embryos were skinned, eviscerated, and dehydrated in 95% ethanol overnight. The mice were transferred to acetone for another 24 hours to dissolve fat tissue, after which the tissue was stained with Alcian blue 8GX (0.03%, m/v in 70% ethanol) and Alizarin red S (0.005%, m/v in dH_2_O) solution containing 10% acetic acid and 80% ethanol. The stained skeletons were cleared in 1% KOH followed by a gradient of glycerol (30%, 50%, and 80%).

### Serum analysis.

Mouse blood samples were collected through cardiac puncture after 3 hours of fasting. Blood samples were allowed to clot for 2 hours at room temperature before centrifugation for 15 minutes at room temperature at approximately 1,000*g*. Freshly prepared serum samples were stored at –80°C for later use. Serum P1NP, OCN, and CTX-I levels were measured by using P1NP ELISA kit, OCN ELISA kit, and CTX-I ELISA kit, respectively, according to the manufacturer’s instructions.

### Cell culture and transfection.

Primary murine calvarial cells were isolated as follows. The calvaria was dissected out from P3 pups; it was then cleaned and chopped into small pieces. Four sequential 10-minute digestions were performed with 1 mg/mL collagenase P in a shaking incubator at 37°C. The first digestion was discarded, and the rest were combined. The digested calvarial cells were centrifuged at 300*g* for 5 minutes at room temperature and plated with α-MEM supplemented with 15% FBS. The cells were seeded at 50,000 cells/mL. α-MEM with 10% FBS, 50 mg/mL ascorbic acid and 10 mM β-glycerophosphate was used to initiate terminal osteoblast differentiation. At day 7, RNA was isolated and alkaline phosphatase staining was performed using the 1-step nitro-blue tetrazolium (NBT) and 5-bromo-4-chloro-3′-indolyphosphate p-toluidine salt (BCIP) solution. At day 14, alizarin red and von Kossa staining was performed to visualize matrix mineralization.

Primary bone cells migrated from cultured bone shaft were isolated as follows. Adult long bones were isolated, and all connective tissue was removed. The epiphyses were cut off, and marrow was removed by centrifugation at 8,000*g* for 10 seconds at room temperature. Bone tissue was then chopped into small pieces and plated with α-MEM supplemented with 15% FBS. Three to 5 days later, migrated bone cells were used for experiments as needed. In separate experiments, HEK293 cells were transfected with DDK-tagged RUNX2 expression plasmid using a FuGENE transfection reagent according to the manufacturer’s recommendations.

### CRISPR/Cas9-mediated gene KO.

Lentiviral vectors expressing single-guide RNAs (sgRNA) targeting either *Gclc*, *Gclm*, *Gls*, or Luciferase and mCherry were cloned into the LentiGuide-Puro plasmid according to the previously published protocol (73). Then, sgRNA-carrying lentivirus infected the primary calvarial cells isolated from *Rosa26-Cas9*–knockin mice, which ubiquitously and constitutively express Cas9 endonuclease under the direction of a CAG promoter. The LentiGuide-Puro plasmid was a gift from Feng Zhang (Massachusetts Institute of Technology, Cambridge, Massachusetts, USA) (Addgene plasmid 52963). Sequences of each sgRNA protospacer are shown in Supplemental Table 5.

### Virus production for sgRNA KO.

To make a virus, the indicated vector was cotransfected with plasmids pMD2.g and psPax2 into HEK293 cells. After 48 hours, virus-containing media were collected and filtered through a 0.45 μm filter. For infection, calvarial cells were infected at 50% confluency for 24 hours followed by recovery in regular media for another 24 hours. Then, selection was performed by puromycin (5 μg/mL) or blasticidin S-HCl (10 μg/mL) for 48 hours.

### Reduced and oxidized GSH LC-MS/MS analysis.

Cells were incubated with either 2 mM [U-13C] glutamine for 6 hours or 1.33 mM [U-13C] glycine for 12 hours. Cells were washed with cold PBS and extracted twice with 250 μL cold methanol containing 10 mM N-Ethylmaleimide on dry ice. The extracts were sonicated for 1 minute and then centrifuged for 15 minutes (13,000*g* at 4°C). The supernatant was completely dried with N_2_ gas. The dried residue was redissolved in 50 μL H_2_O and transferred to LC-MS vials for analysis. Briefly, LC-MS/MS was run with a Sciex QTRAP 6500^+^ MS connected with a Sciex AD UHPLC. A Phenomenex Aeris PEPTIDE 1.7 μM XB-C18 column (50 × 2.1 mm) was employed for separation at 46°C with a flow rate at 0.3 mL/min. A gradient method was conducted with 2 mobile phases. Mobile phase A was 98% H_2_O and 2% acetonitrile containing 0.1% formic acid. Mobile phase B was 98% acetonitrile and 2% H_2_O containing 0.1% formic acid. The gradient started with 100% A for the first 2 minutes and increased B to 40% at 4.2 minutes. B was further increased to 95% at 7.2 minutes and was maintained at 95% for 3.8 minutes and returned to initial condition within 1 minute. Finally, the column was reequilibrated for 8 minutes with the initial condition before the next injection. The injection volume was 1 μL. Multiple reaction monitoring in positive mode was used for GSH assay. The MS/MS parameters were set as following: curtain gas, 35 psi; source temperature, 600°C; Gas 1, 55 psi; Gas 2, 55 psi; CAD, 10 V; ion spray voltage, 5500 V; entrance potential, 10 V; and collision cell exit potential, 14 V. DP and CE were set at 45 V and 26 V for GSH. DP and CE were 140 V and 32 V for oxidized GSH (GSSG).

### Proteomic analysis.

Naive calvarial cells were cultured with or without 250 μM H_2_O_2_ for 24 hours. Cells were then washed, scraped, and lysed in RIPA buffer. Protein samples were loaded onto 10% SDS-PAGE gels and run 10 mm into resolving gel. Gels were stained with Coomassie blue, and gel bands were cut out and diced into 1 mm cubes. Proteomic analysis was performed by UT Southwestern Proteomics Core.

### GLS activity assay.

Cells were washed 3 times with HBSS and cultured for 20 minutes in α-MEM media containing 5 mM glucose, 2 mM Glutamine, and 4 μCi/mL L-(2,3,4-^3^H)-Glutamine. GLS activity was terminated by washing 3 times with ice-cold HBSS. Cells were scraped into 1 mL ice-cold Milli-Q water and sonicated for 1 minute with 1 second pulses at 20% amplitude. After clarification, cell lysates were bound onto AG 1-X8 polyprep anion exchange columns. Uncharged glutamine was eluted with three 2 mL volumes of H_2_O. Glutamate and downstream metabolites were eluted with three 2 mL volumes of 0.1 M HCl. Eluent fractions were pooled and combined with 4 mL scintillation cocktail, and DPM was measured using a Beckman LS6500 Scintillation counter.

### ROS measurement.

Intracellular ROS levels were measured by means of flow cytometry using a cell-permeable oxidation-sensitive fluorescent probe dye, chloromethyl derivative of 2′,7′-dichlorodihydrofluorescin diacetate (CM-H2DCFDA). Briefly, cells were washed with PBS, trypsinized, counted, and resuspended at 1 × 10^6^ cells/mL in ice-cold PBS containing 5 μM CM-H2DCFDA. After incubation for 30 minutes at 37°C, cells were then washed with PBS 3 times before collection by centrifugation at 400*g* for 5 minutes at room temperature. Cells were resuspended in 500 μL ice-cold PBS, followed by propidium iodide staining for 5 minutes. The mean fluorescence intensity of the DCF was measured by a FACSCanto II flow cytometer (BD Biosciences). Data were analyzed using FlowJo (version 10).

### Measurement of CAT activity.

CAT activity of bone extracts was measured by a commercial Fluorimetric CAT Assay Kit according to the manufacturer’s instructions.

### TUNEL assay.

TUNEL staining was performed using the In Situ Cell Death Detection Kit. Briefly, cells were fixed in 4% paraformaldehyde for 15 minutes and then permeabilized by Triton X-100 (0.1%, v/v in 0.1% sodium citrate) for 2 minutes on ice. The permeabilized cells were then incubated with TUNEL reaction mixture for 60 minutes at 37°C.

### RNA isolation and qPCR.

Total RNA from bone tissue or cells was extracted using Trizol reagent following a standard RNA extraction protocol. First-strand cDNA was synthesized from 500 ng of total RNA with the iScript cDNA synthesis kit. qPCR was performed in technical and biological triplicates in a 96-well format on an Applied Biosystems QuantStudio 3, using SYBR Green chemistry. The PCR programs were 95°C for 3 minutes followed by 40 cycles of 95°C for 10 seconds and 60°C for 30 seconds. Gene expression was normalized to *Actb* mRNA, and relative expression was calculated using the 2^−(ΔΔCt)^ method. Primers were used at 0.1 μM, and their sequences are listed in Supplemental Table 6.

### Western blotting.

Cells were lysed in RIPA buffer containing 50 mM Tris–HCl (pH 7.4), 150 mM NaCl, 1% NP-40, 0.5% sodium deoxycholate, and 0.1% SDS supplemented with protease inhibitor cocktail and phosphatase inhibitors. For isolating protein from bone tissue, femurs were freshly dissected and washed in ice-cold PBS. Both ends of the femur were cut off, and BM was flushed out by centrifugation at 8,000*g* for 10 seconds at room temperature. Then, bone shaft was vigorously chopped 100 times in RIPA buffer. Protein fractions were collected by centrifugation at 15,000*g* at 4°C for 10 minutes. Protein concentration was estimated by the BCA method. Protein samples were then mixed with 4***×*** loading buffer and boiled for 10 minutes. Then, 25 μg of total protein was loaded onto 4%–15% polyacrylamide gel and then transferred onto polyvinylidene difluoride (PVDF) membranes. The membranes were blocked for 1 hour at room temperature with 5% milk in TBST (TBS, 0.1% Tween20) and then incubated with specific primary antibodies overnight at 4°C. The following primary antibodies were used in this study: anti-GCLC, anti-GLS, anti-RUNX2, anti-PRDX1-SO_3_, anti-PRDX1, anti-NQO1, anti-CAT, anti-ATF4, anti–collagen I, anti-Flag, Normal rabbit IgG isotype control antibody, anti-Biotin, and anti–β-actin. All primary antibodies were diluted 1:1,000 in TBST containing 5% milk. Membranes were then washed 3 times using TBST and further incubated with HRP-linked secondary antibody, anti-rabbit or anti-mouse, in 5% milk for 1 hour at room temperature. All blots were developed using either the Clarity ECL substrate or the Super Signal West Femto substrate. Each experiment was repeated for at least 3 times.

### DCP-Bio1 labeling.

Cells were scraped and lysed, and they reacted with the cysteine sulfenic acid probe DCP-Bio1 in a modified lysis buffer containing 50 mM Tris-HCl (pH 7.5), 100 mM NaCl, 0.1% SDS, 0.5% sodium deoxycholate, 0.5% NP-40, 0.5% Triton X-100, 50 mM NaF, 1 mM PMSF, 1 mM DCP-Bio1, 100 μM diethylene triamine pentaacetic acid, 10 mM N-ethylmaleimide, 10 mM iodoacetamide, 200 U/mL CAT, and protease inhibitor cocktail. The cell lysates were incubated on ice for 1.5 hour and then centrifuged at 9,600*g* for 10 minutes at 4°C. Unreacted DCP-Bio1 in the supernatant was removed using Bio-Rad P6-Spin Columns following manufacturer’s instructions. General biotin-labeled oxidized proteins were visualized by Western blotting using Streptavidin-HRP. For identifying specific oxidized protein, the cell lysates were used for immunoprecipitation experiment.

### Immunoprecipitation.

The immunoprecipitation assay was performed using the Pierce Classic Magnetic IP/Co-IP Kit according to the manufacturer’s instructions. Briefly, cells were lysed with ice-cold IP lysis buffer containing protease/phosphatase inhibitors. The whole-cell lysates were centrifuged at 13,000*g* for 10 minutes at 4°C to remove cell debris. Protein concentration was measured by the BCA method. Whole cell lysates containing 500 μg of total proteins were incubated with 5 μg of desired IP antibodies overnight at 4°C with rotation to form the immune complex. The immune complex was then captured by incubating with 0.25 mg Pierce Protein A/G Magnetic Beads at room temperature for 1 hour. The beads were collected on a magnet stand and rigorously washed 3 times using IP wash buffer. Target protein was eluted at room temperature for 10 minutes using elution buffer. Immunoprecipitates or whole-cell lysates were then resolved by Western blotting.

### Statistics.

Statistical analyses were performed using GraphPad Prism 6 software. All data are represented as mean ± SD. In cell culture studies, statistical significance was determined by 2-tailed Student’s unpaired *t* test. For animal studies, statistical significance was determined by 2-tailed Student’s paired *t* test comparing paired littermate controls or 2-way ANOVA. *P* < 0.05 was considered statistically significant. All experiments were performed with at least 3 biological replicates. Representative experiments were repeated at least 3 times.

### Study approval.

All mouse procedures were approved by The Animal Studies Committees at Duke University and the University of Texas Southwestern Medical Center.

### Data availability.

Proteomics data are deposited in the UT Southwestern Research Data Repository (https://dataverse.tdl.org/dataset.xhtml?persistentId=doi:10.18738/T8/XJHFWK). All individual data values presented in graphs are available in the Supporting Data Values file.

## Author contributions

CMK designed and supervised the study. GH, YY, DS, SMPM, YR, and CMK performed the experiments. GH, GZ, and CMK analyzed and interpreted the data. SMPM designed, validated, and provided the sgRNAs. GZ conducted and analyzed the LC-MS/MS. GH and CMK wrote the manuscript. All authors approved the final version of the manuscript.

## Supplementary Material

Supplemental data

Supporting data values

## Version 1 07/11/2023

In-Press Preview

## Version 2 08/22/2023

Electronic publication
