# Supplementary material for: Glutathione limits RUNX2 oxidation and degradation to regulate bone formation
Source: JCI Insight. 2023 Aug 22;8(16):e166888. doi: 10.1172/jci.insight.166888 (PMC10543723; doi:10.1172/jci.insight.166888)
Supplement: Supplemental data [file jciinsight-8-166888-s183.pdf]

Supplemental information

Supplemental information includes 8 figures and 6 tables

Key resources table

REAGENT or RESOURCE	SOURCE	IDENTIFIER
Antibodies		
Anti-Osteocalcin	Millipore	Cat# AB10911; RRID: AB_1587337
Anti-8-Hydroxy-deoxyguanosine	Abcam	Cat# ab48508; RRID: AB_867461
Anti-GCLC	Abcam	Cat# ab53179; RRID: AB_880163
Anti-PRDX1-SO_3	Abcam	Cat# ab16830; RRID: AB_443491
Anti-PRDX1	Abcam	Cat# ab41906; RRID: AB_2284360
Streptavidin-HRP	Abcam	Cat# ab7403
Anti-GLS	Cell Signaling Technology	Cat# 56750
Anti-RUNX2	Cell Signaling Technology	Cat# 12556; RRID: AB_2732805
Anti-NQO1	Cell Signaling Technology	Cat# 62262; RRID: AB_2799623
Anti-Catalase	Cell Signaling Technology	Cat# 14097; RRID: AB_2798391
Anti-Flag	Cell Signaling Technology	Cat# 14793; RRID: AB_2572291
Anti-Cleaved Caspase3	Cell Signaling Technology	Cat# 9664; RRID: AB_2070042
Anti-Caspase3	Cell Signaling Technology	Cat# 9662; RRID: AB_331439
Normal rabbit IgG	Cell Signaling Technology	Cat# 2729; RRID: AB_1031062
Anti-β-actin	Cell Signaling Technology	Cat# 4970; RRID: AB_2223172
Anti-ATF4	Santa Cruz	Cat# sc-200; RRID: AB_2058752
Anti-Collagen I	Santa Cruz	Cat# sc-293182; RRID: AB_2797597
Anti-HRP goat anti-rabbit	Cell Signaling Technology	Cat# 7074; RRID: AB_2099233
Anti-HRP anti-mouse	Cell Signaling Technology	Cat# 7076; RRID: AB_330924
Chemicals, peptides, and recombinant proteins		
Ascorbic acid	Sigma	Cat# A4544
β-glycerophosphate	Sigma	Cat# G9422
L-glutamine	Sigma	Cat# G7513
BPTES	Sigma	Cat# SML0601
BSO	Sigma	Cat# B2515
Erastin	Sigma	Cat# E7781
Glutathione reduced ethyl ester	Sigma	Cat# G1404
Dimethyl α-ketoglutarate	Sigma	Cat# 349631
L-Glutamic acid dimethyl ester	Sigma	Cat# 49560
Catalase−polyethylene glycol	Sigma	Cat# C4963
N-Acetyl-L-cysteine	Sigma	Cat# A9165
(U-^{13}C)-Glutamine	Sigma	Cat# 605166
(U-^{13}C)-Glycine	Sigma	Cat# 279439
L-(2,3,4-^{3}H)-Glutamine	American Radiolabeled Chemicals	Cat# ART0149a
DCP-Bio1	Millipore	Cat# NS1226
Catalase from bovine liver	Sigma	Cat# C30
CM-H2DCFDA	Thermo Fisher	Cat# C6827
Cycloheximide	Sigma	Cat# C7698
MG-132	Sigma	Cat# M7449
Hydrogen peroxide	Fisher Scientific	Cat# H325-4
Collagenase P	Roche	Cat# 11213873001
Proteinase K	Sigma	Cat# 1.24568
FuGENE 6	Promega	Cat# E269A
Alizarin-3-methyliminodiacetic acid	Sigma	Cat# A3882
Calcein	Sigma	Cat# C0875
Alizarin red S	Sigma	Cat# A5533
Alcian blue 8GX	Sigma	Cat# A3157
Silver Nitrate	Lab Pro Inc.	Cat# 6830-4
Trizol	Thermo Fisher	Cat# 15596018
Triton X-100	Sigma	Cat# T8787
Puromycin	Thermo Fisher	Cat# A11138-03
Blasticidin S-HCl	Thermo Fisher	Cat# A11139-03
N-Ethylmaleimide	Sigma	Cat# E1271
Trypsin-EDTA	Thermo Fisher	Cat# 25200-072
Fetal bovine serum	Thermo Fisher	Cat# 16000-044
Protease inhibitor cocktail	Roche	Cat# 11697498001
Phosphatase inhibitor	Roche	Cat# 04906837001

(*continued on next page*)

REAGENT or RESOURCE	SOURCE	IDENTIFIER
Critical Commercial Assays		
In-situ Cell Death Detection Kit	Roche	Cat# 11684795910
One-step NBT/BCIP solution	Thermo Fisher	Cat# PI34042
Clarity ECL substrate	Bio-Rad	Cat# 1705060
Super Signal West Femto substrate	Thermo Fisher	Cat# PI34095
iScript cDNA Synthesis Kit	Bio-Rad	Cat# 1708841
Mouse P1NP ELISA Kit	Immunodiagnostic Systems	Cat# AC-33F1
Mouse CTX-I ELISA Kit	Immunodiagnostic Systems	Cat# AC-02F1
Mouse Osteocalcin ELISA Kit	ABclonal	Cat# RK03088
Fluorimetric Catalase Assay Kit	AAT Bioquest	Cat# 11306
Pierce Classic Magnetic IP/Co-IP Kit	Thermo Fisher	Cat# 88804
Experimental Models: Cell Lines		
HEK293T	ATCC	Cat# CRL-3216
Experimental Models: Organisms/Strains		
Mouse: $Gclc^{tm1a(EUCOMM)Wtsi}$	European Mouse Mutant Archive	RRID: IMSR_EM:05085
Mouse: $Gls^{fl/fl}$: $Gls^{tm2.1Sray}/J$	The Jackson Laboratory	RRID: IMSR_JAX:017894
Mouse: $Rosa26^{Cas9}$	The Jackson Laboratory	RRID: IMSR_JAX:024858
Mouse: $Rosa26^{FLP1}$	The Jackson Laboratory	RRID: IMSR_JAX:003946
Mouse: *MitoCat*	The Jackson Laboratory	RRID: IMSR_JAX:030712
Mouse: *Sp7-tTA;tetO-EGFP/Cre*	The Jackson Laboratory	RRID: IMSR_JAX:006361
Mouse: $Runx2^{+/-}$	The Karsenty Lab	N/A
Mouse: *C57Bl/6J*	The Jackson Laboratory	RRID: IMSR_JAX:000664
Oligonucleotides		
See Table S5 for sgRNA sequences	Washington University in St. Louis	N/A
See Table S6 for qPCR primers	https://www.idtdna.com/	N/A
Recombinant DNA		
DDK-RUNX2 plasmid	OriGene	Cat# MR227321
pMD2.G	Addgene	Cat# 12259
psPax2	Addgene	Cat# 12260
Software and Algorithms		
Image J	https://imagej.nih.gov/ij/	N/A
Graphpad Prism 6	https://www.graphpad.com/	N/A
Other		
α-MEM	Thermo Fisher	Cat# 12561-056
Glutamine Free α-MEM	Corning	Cat# 15-012-cv
AG 1-X8 Anion Exchange Column	Bio-Rad	Cat# 7316212
Bio-Rad P6-Spin Column	Bio-Rad	Cat# 7326227
SYBR Green	Bio-Rad	Cat# 1725275
PicoLab Rodent Diet 290	LabDiet	Cat# 5053

Figure S1. Related to Figure 1

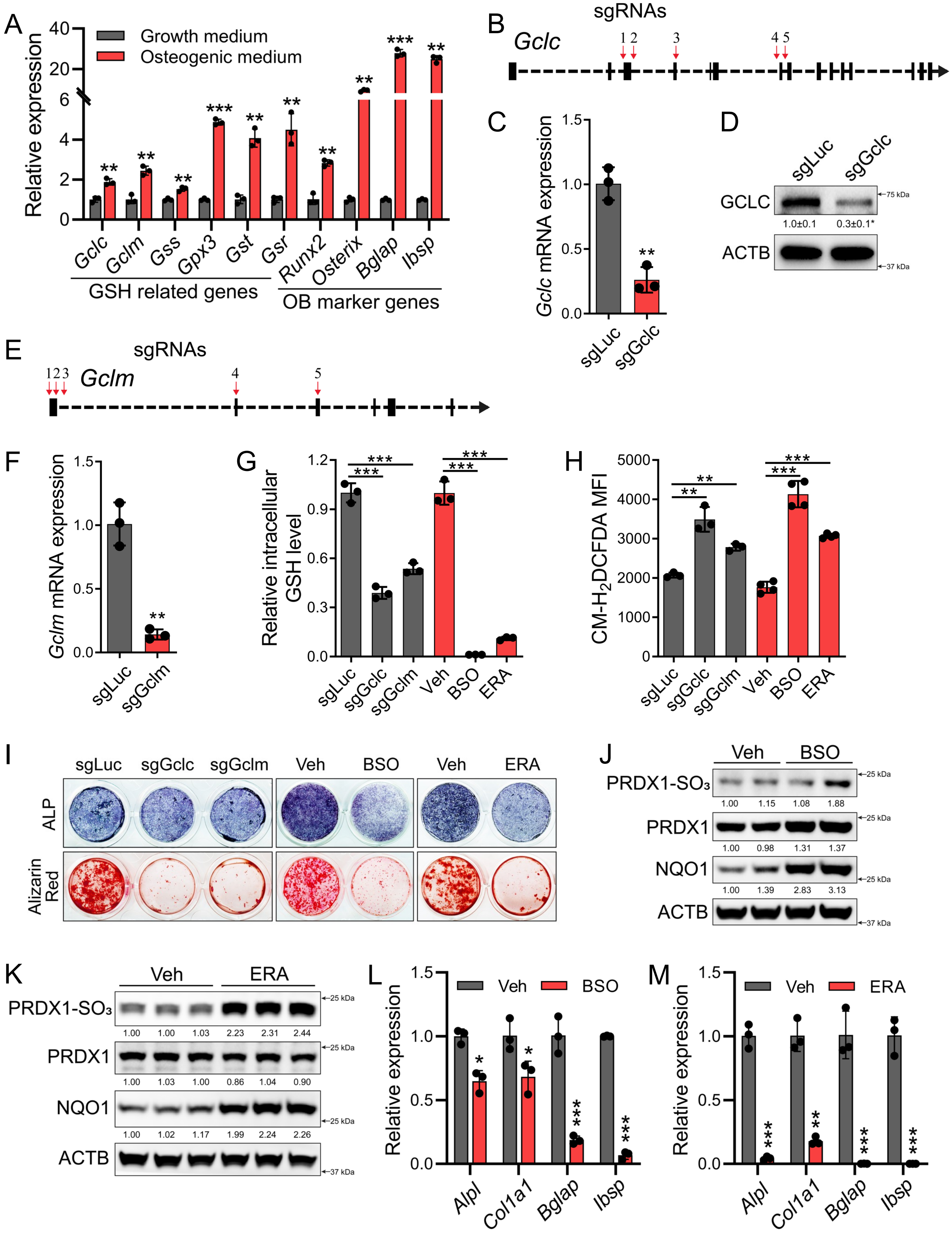

Figure S1. Related to Figure 1. (**A**) qPCR analysis of GSH pathway related genes and osteogenic marker genes in naïve and differentiated calvarial cells (*n* = 3). **(B-F)** Crispr targeting strategy and qPCR and western blot analysis of the effect of *Gclc* and *Gclm* targeting (*n* = 3). (**G-H**) Intracellular GSH levels (**G**) and ROS levels (**H**) in naïve calvarial cells with indicated treatments (*n* = 3-4). (**I**) Representative images of alkaline phosphatase (ALP) and alizarin red staining of differentiated calvarial cells with indicated treatments. (**J-K**) Western blot analysis of PRDX1-SO_3, PRDX1, and NQO1 in naïve calvarial cells treated with BSO (**J**) or Erastin (**K**) (*n* = 2-3). PRDX1-SO3 was normalized to total PRDX1; all other proteins were normalized to ACTB. (**L-M**) qPCR analysis of osteogenic marker genes in calvarial cells differentiated in media containing either BSO or ERA (*n* = 3). Data represent mean ± SD. *$P < 0.05$, **$P < 0.01$, ***$P < 0.001$. Two-tailed Student's unpaired *t* test.

Figure S2. Related to Figure 1

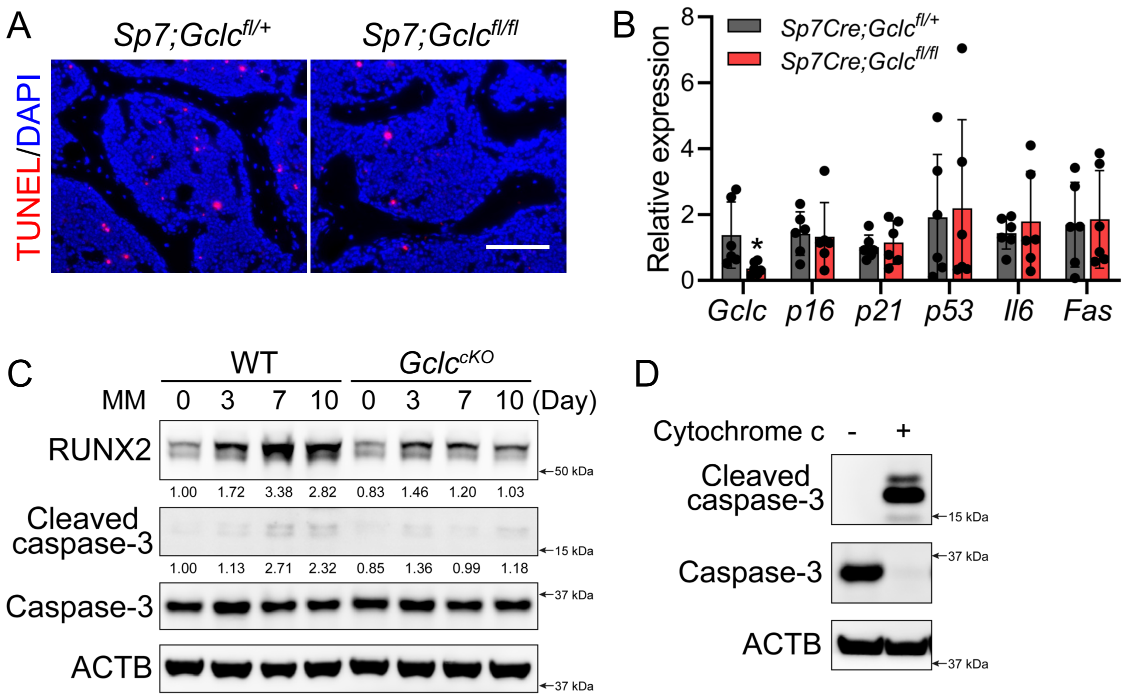

Figure S2. Related to Figure 1. (**A**) Representative histological sections showing TUNEL staining performed in distal femurs from *Sp7Cre;Gclc*$^{fl/+}$ and *Sp7Cre;Gclc*$^{fl/fl}$ mice (n = 8). Scale bar: 100 µm. (**B**) qPCR analysis of senescence and apoptosis marker genes in bone tissues isolated from *Sp7Cre;Gclc*$^{fl/+}$ and *Sp7Cre;Gclc*$^{fl/fl}$ mice (n = 6). (**C**) Western blot analysis of RUNX2, cleaved caspase-3, and caspase-3 in WT and *Gclc*^{cKO} calvarial cells during osteogenic differentiation (n = 3). Cleaved caspase-3 was normalized to total caspase-3. (**D**) Western blot analysis of cleaved caspase-3 and caspase-3 in calvarial cells treated with or without Cytochrome c. Data represent mean ± SD. *P < 0.05, **P < 0.01, ***P < 0.001. Two-tailed Student's paired t test.

Figure S3. Related to Figure 2

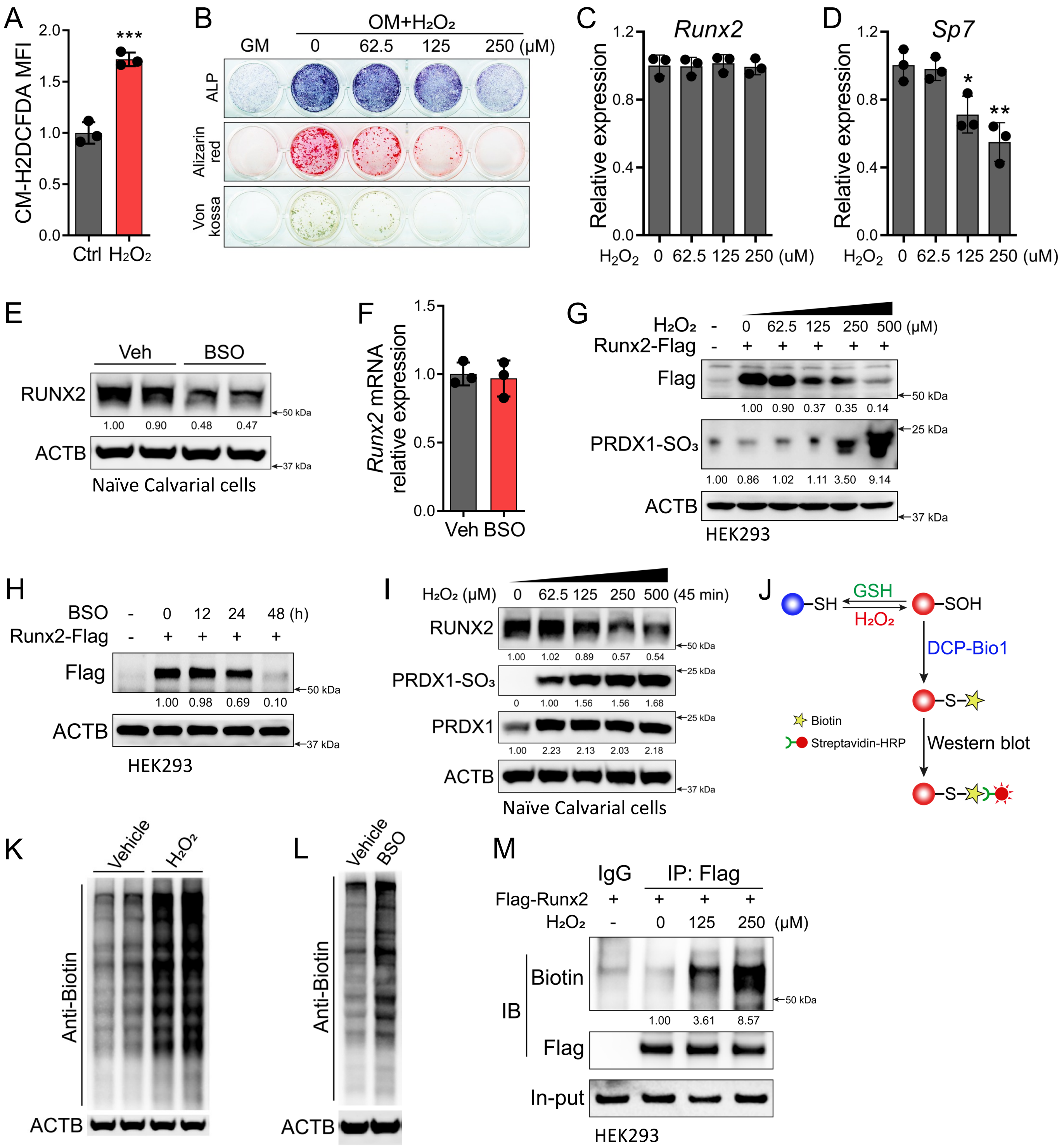

Figure S3. Related to Figure 2. (**A**-**B**) Effects of H_2O_2 on ROS levels (**A**) or functional assays of osteoblast differentiation (**B**) (*n* = 3). (**C**-**D**) qPCR analysis of *Runx2* (**C**) and *Sp7* (**D**) mRNA expression in calvarial cells exposed to a gradient of H_2O_2 (*n* = 3). (**E**-**F**) The effect of BSO on RUNX2 protein (**E**) or mRNA expression (**F**) in calvarial cells (*n* = 2-3). (**G**-**H**) Western blot analysis of the effect of H_2O_2 (**G**) or BSO treatment (**H**) on Flag-RUNX2 or PRDX1-SO_3 in HEK293 cells. (**I**) Western blot analysis of RUNX2, PRDX1-SO_3, and PRDX1 in calvarial cells exposed to a gradient of H_2O_2 for 45 minutes. PRDX1-SO3 was normalized to total PRDX1; all other proteins were normalized to ACTB. **(J)** Schematic illustrating the working principle of cysteine sulfenic acid probe (**K-L**) Western blot analysis of DCP-Biotin incorporation into total proteins in calvarial cells treated with H_2O_2 (**K**) or BSO (**L**) (*n* = 2-3). (**M**) Western blot analysis of immunoprecipitated Flag-tagged RUNX2 from H_2O_2-treated HEK293 cells. Normal rabbit IgG was used as a negative control. Data represent mean ± SD. $^*P < 0.05$, $^{**}P < 0.01$, $^{***}P < 0.001$. Two-tailed Student's unpaired *t* test.

Figure S4. Related to Figure 2

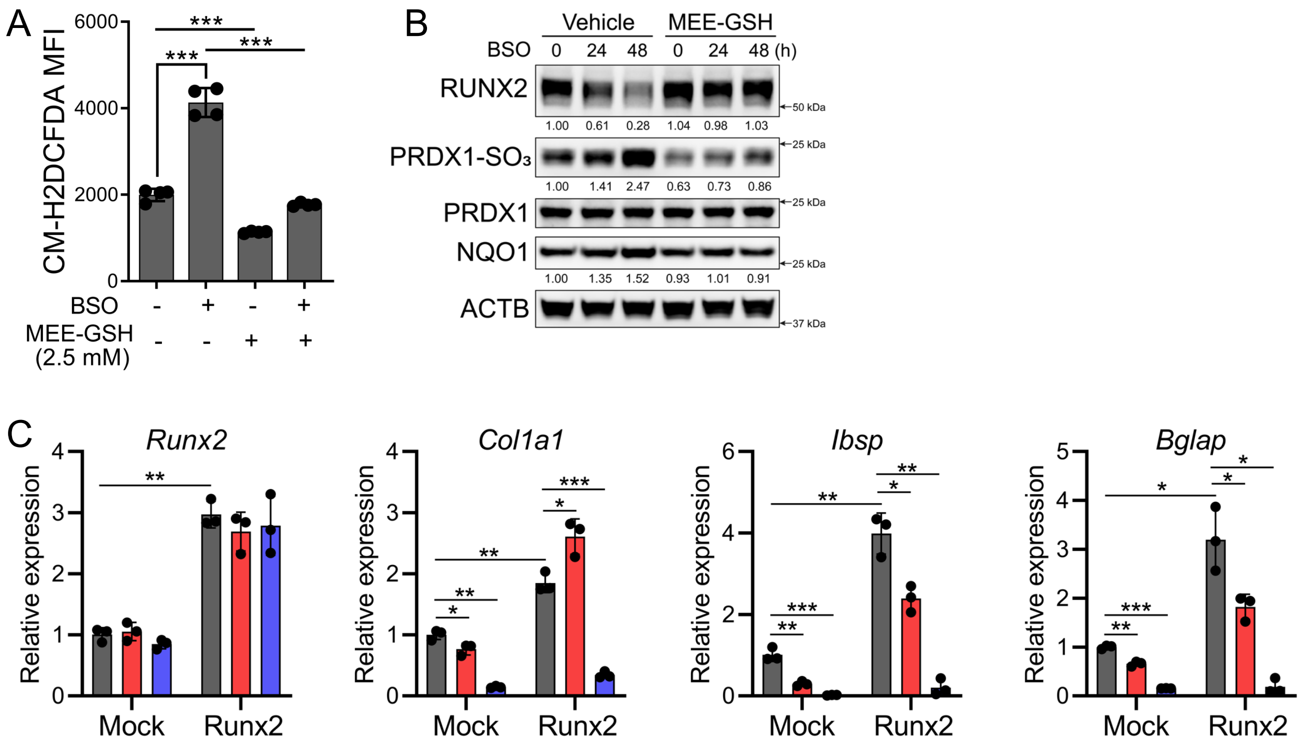

Figure S4. Related to Figure 2. (**A**-**B**) Effects of BSO and MEE-GSH treatment on ROS levels (**A**) or protein expression determined by western blot (**B**) (*n* = 3-4). PRDX1-SO3 was normalized to total PRDX1; all other proteins were normalized to ACTB. MEE-GSH, Glutathione mono-ethyl ester. (**C**) *Runx2* and osteogenic marker gene expression in calvarial cells transfected with or without Flag-Runx2 under different doses of H_2O_2 ($n = 3$). Data represent mean ± SD. *$P < 0.05$, **$P < 0.01$, ***$P < 0.001$. Two-tailed Student's unpaired *t* test.

Figure S5. Related to Figure 3

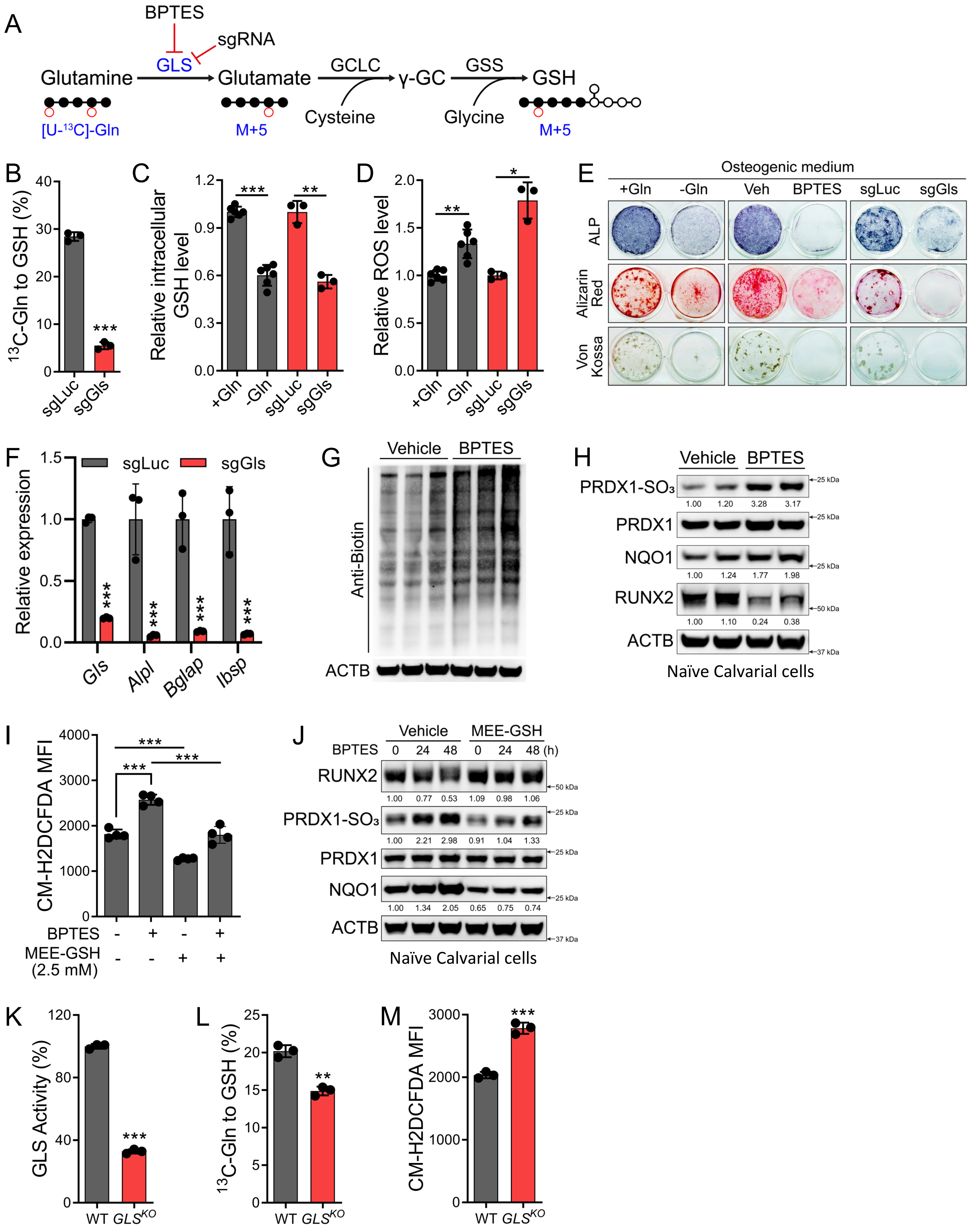

Figure S5. Related to Figure 3. (**A**) Graphical depiction of GSH biosynthesis and the route of [U-^{13}C]glutamine carbon into GSH. (**B**) Effect of GLS targeting on the fractional contribution of [U-^{13}C]glutamine to GSH (*n* = 3). (**C-F**) Effect of limiting glutamine availability or inhibiting glutamine metabolism on intracellular GSH (**C**), ROS levels (**D**), functional assays of osteoblast differentiation (**E**), or expression of osteoblast marker genes by qPCR (**F**) in calvarial cells treated as indicated (*n* = 3-6). (**G**) Effect of BPTES treatment on the incorporation of DCP-Biotin into total protein in calvarial cells (*n* = 3). (**H**) Western blot analysis of PRDX1-SO_3, PRDX1, NQO1, and RUNX2 in calvarial cells treated with or without BPTES (*n* = 2). PRDX1-SO3 was normalized to total PRDX1; all other proteins were normalized to ACTB. (**I-J**) Evaluation of the effect of MEE-GSH on ROS levels (**I**) or protein expression of RUNX2, PRDX1-SO_3, PRDX1, and NQO1 (**J**) in BPTES treated calvarial cells (*n* = 4). (**K-M**) Evaluation of the effect of *Gls* knockout on GLS activity (**K**), fractional contribution of [U-^{13}C]glutamine to GSH (**L**) or ROS levels (**M**) in primary bone cells migrated from cultured bone shaft isolated from $Sp7Cre;Gls^{fl/+}$ (WT) or $Sp7Cre;Gls^{fl/fl}$ (GLS^{KO}) mice (*n* = 3). Data represent mean ± SD. **P* < 0.05, ***P* < 0.01, ****P* < 0.001. Two-tailed Student's unpaired *t* test.

Figure S6. Related to Figure 4

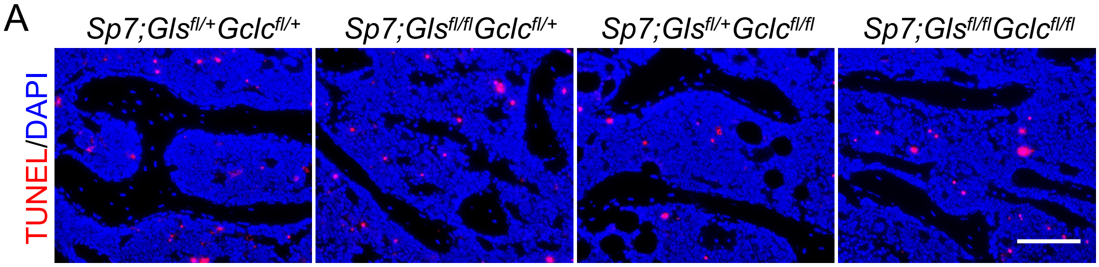

Figure S6. Related to Figure 4. (**A**) TUNEL staining of distal femurs isolated from *Sp7Cre;Gls*$^{fl/+}$*Gclc*$^{fl/+}$, *Sp7Cre;Gls*$^{fl/fl}$*Gclc*$^{fl/+}$, *Sp7Cre;Gls*$^{fl/+}$*Gclc*$^{fl/fl}$, and *Sp7Cre;Gls*$^{fl/fl}$*Gclc*$^{fl/fl}$ mice ($n = 4$). Scale bar: 100 μm. Data represent mean ± SD. *$P < 0.05$, **$P < 0.01$, ***$P < 0.001$. Two-tailed Student's unpaired *t* test.

Figure S7. Related to Figure 5

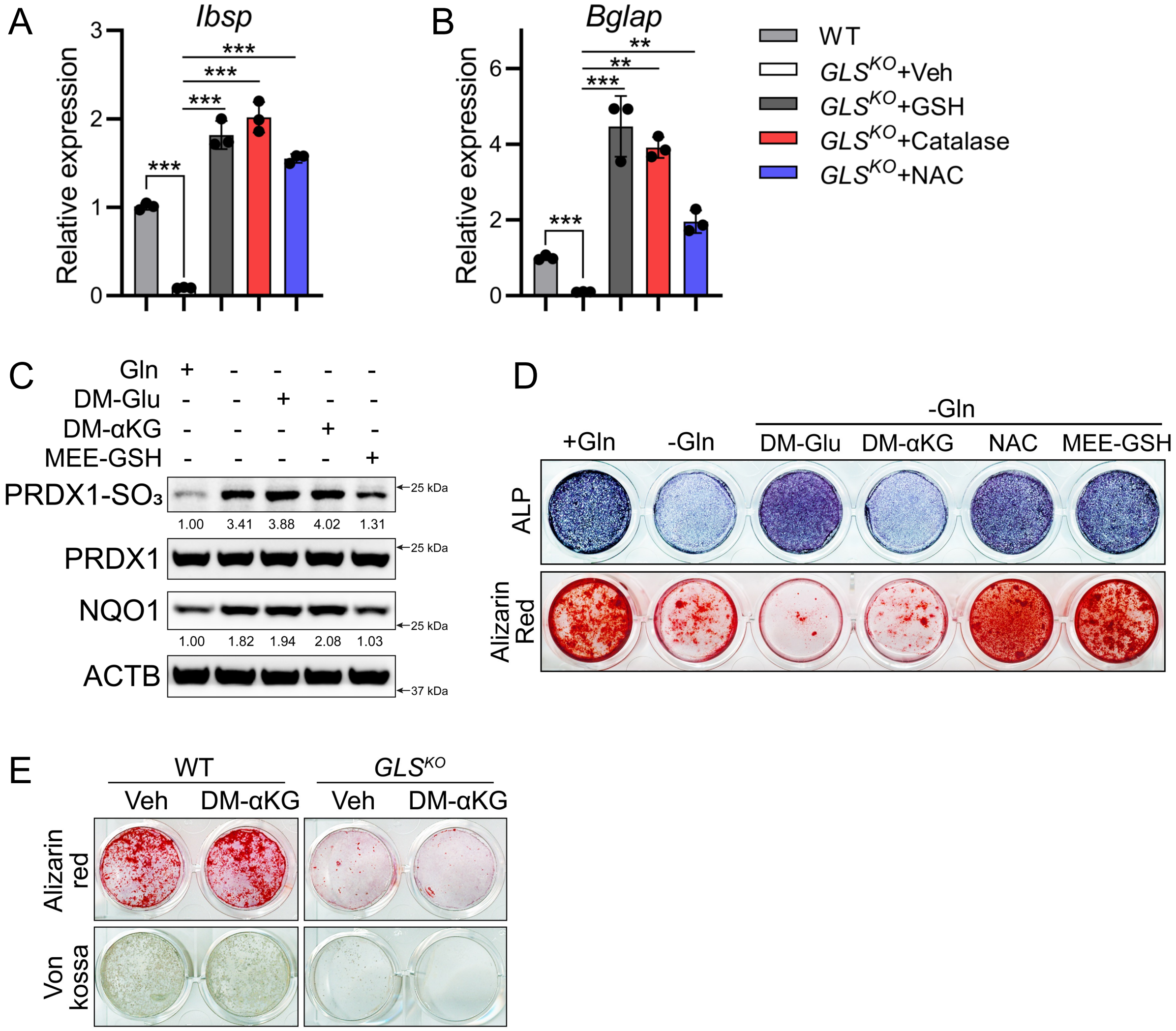

Figure S7. Related to Figure 5. (**A**-**B**) qPCR analysis of *Ibsp* (**A**) and *Bglap* (**B**) expression in calvarial cells isolated from $Sp7Cre;Gls^{fl/+}$ (WT) or $Sp7Cre;Gls^{fl/fl}$ (GLS^{KO}) mice and induced to undergo osteoblast differentiation in media containing the indicated antioxidants (n = 3). (**C**) Western blot analysis of PRDX1-SO_3, PRDX1, and NQO1 in calvarial cells supplemented with dimethyl-glutamate (DM-Glu), dimethyl-α-ketoglutarate (DM-α-KG), or mono-ethyl ester-GSH (MEE-GSH) in the absence of glutamine (n = 2). PRDX1-SO3 was normalized to total PRDX1; all other proteins were normalized to ACTB. (**D**) ALP and alizarin red staining of differentiated calvarial cells supplemented with dimethyl-glutamate (DM-Glu), dimethyl-α-ketoglutarate (DM-α-KG), N-acetylcysteine (NAC), or mono-ethyl ester-GSH (MEE-GSH) in the absence of glutamine (n = 3). (**E**) Alizarin red and von kossa staining of differentiated WT and GLS^{KO} calvarial cells cultured with or without DM-α-KG (n = 3). Data represent mean ± SD. *$P < 0.05$, **$P < 0.01$, ***$P < 0.001$. Two-tailed Student's unpaired *t* test.

Figure S8. Related to Figure 6

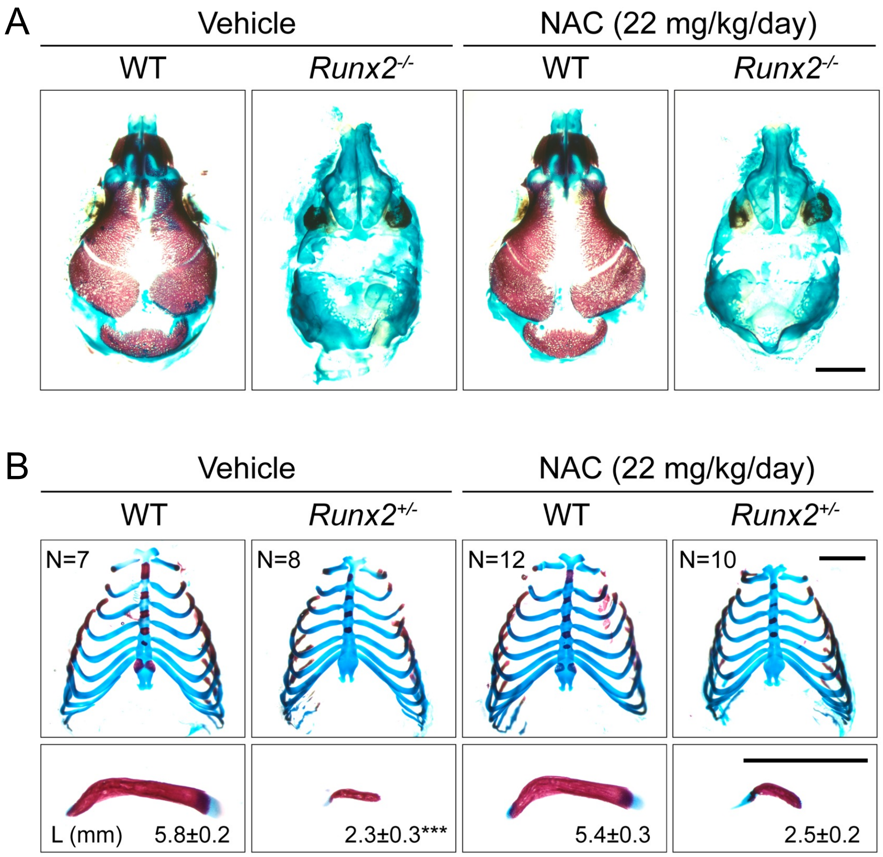

Figure S8. Related to Figure 6. (**A**) Alcian blue/alizarin red staining of the skull of E18.5 wild type or *Runx2*$^{-/-}$ littermate embryos carried by NAC- or vehicle-treated mothers (n = 6-10). Scale bar: 2 mm. (**B**) Alcian blue/alizarin red staining of clavicle and sternum with ribs of E18.5 wild type or *Runx2*$^{+/-}$ littermate embryos carried by NAC- or vehicle-treated mothers (n = 7-12). Scale bar: 2 mm. Data represent mean ± SD. $^{*}P < 0.05$, $^{**}P < 0.01$, $^{***}P < 0.001$. Two-way ANOVA test.

Supplementary table 1. Bone parameters of *Sp7Cre;Gclc*$^{fl/fl}$ mice at 6 months of age

	Sp7Cre;Gclc$^{fl/+}$	*Sp7Cre;Gclc*$^{fl/fl}$
**Male (*n*)**	8	8
Tb.BV/TV (%)	13.71±2.56	9.90±1.86**
Tb.N (1/mm)	4.51±0.24	4.04±0.38*
Tb.Th (mm)	0.045±0.003	0.041±0.005
Tb.Sp (mm)	0.22±0.01	0.24±0.03
BMD (mg HA/ccm)	177±26	148±21*
Ct.BV/TV (%)	28.90±1.88	29.32±1.57
Ct.Th (mm)	0.190±0.009	0.174±0.010*
T.Ar (mm^2)	2.15±0.18	1.98±0.19*
**Female (*n*)**	7	7
Tb.BV/TV (%)	4.90±0.57	3.30±0.67**
Tb.N (1/mm)	3.92±0.26	3.62±0.34*
Tb.Th (mm)	0.035±0.002	0.032±0.002*
Tb.Sp (mm)	0.25±0.01	0.29±0.02**
BMD (mg HA/ccm)	99±7	83±11*
Ct.BV/TV (%)	28.89±0.70	29.46±1.39
Ct.Th (mm)	0.159±0.009	0.162±0.005
T.Ar (mm^2)	1.53±0.07	1.53±0.06
**Serum Analysis (Male, *n* = 8)**		
P1NP (ng/ml)	63.97±6.73	44.01±11.55**
OCN (ng/ml)	2.68±0.40	2.05±0.45*
CTX-I (ng/ml)	25.55±12.13	23.19±7.11
**Dynamic Histomorphometry (Male, *n* = 4)**		
MS/BS (%)	32.2±2.4	21.8±5.0**
MAR (μm^2/day)	1.95±0.31	1.28±0.24**
BFR($\mu m^3/\mu m^2$/day)	0.62±0.09	0.28±0.09***
**Static Histomorphometry (Male, *n* = 4)**		
Oc.N/BS (1/mm)	3.79±1.22	3.97±0.69
Ob.N/BS (1/mm)	10.43±1.67	6.04±1.35**

Tb.BV/TV, trabecular bone volume/tissue volume; Tb.N, trabecular number; Tb.Th, trabecular thickness; Tb.Sp, trabecular spacing; BMD, bone mineral density; Ct.BV/TV, cortical BV/TV; Ct.Th, cortical thickness; T.Ar, total area; P1NP, procollagen 1 intact N-terminal propeptide; OCN, osteocalcin; CTX-I, C-terminal telopeptide I; MS/BS, mineralizing surface/bone surface; MAR, mineral apposition rate; BFR, bone formation rate; Oc.N/BS, osteoclast number/bone surface; Ob.N/BS, osteoblast number/bone surface. Data represent mean ± SD. *$P < 0.05$, **$P < 0.01$, ***$P < 0.001$. Two-tailed Student's paired *t* test.

Supplementary table 2. Bone parameters of *Sp7Cre;Gls*$^{fl/fl}$ mice at 4 months of age

	Sp7Cre;Gls$^{fl/+}$	*Sp7cre;Gls*$^{fl/fl}$
**Male (*n*)**	11	12
Weight (g)	28.8±2.4	26.0±4.2
Tb.BV/TV (%)	23.9±3.7	15.6±7.3**
Tb.N (1/mm)	4.03±0.90	3.66±1.49
Tb.Th (mm)	0.10±0.04	0.07±0.01*
Tb.Sp (mm)	0.28±0.07	0.36±0.14
BMD (mg HA/ccm)	282±22	236±48*
Ct.BV/TV (%)	0.49±0.03	0.48±0.03
Ct.Th (mm)	0.24±0.02	0.21±0.03*
T.Ar (mm^2)	2.34±0.26	1.99±0.27*
BMD (mg HA/ccm)	1103±27	1093±27
**Female (*n*)**	8	7
Weight (g)	21.6±1.2	20.1±2.8
Tb.BV/TV (%)	8.1±3.9	4.3±2.9**
Tb.N (1/mm)	2.69±1.00	2.71±1.34
Tb.Th (mm)	0.06±0.01	0.04±0.01*
Tb.Sp (mm)	0.43±0.16	0.47±0.23
BMD (mg HA/ccm)	160±17	132±21**
Ct.BV/TV (%)	0.47±0.01	0.41±0.01*
Ct.Th (mm)	0.19±0.01	0.16±0.01*
T.Ar (mm^2)	1.73±0.03	1.60±0.07*
BMD (mg HA/ccm)	1111±20	1087±20
**Dynamic Histomorphometry (Male, *n* = 4)**		
MS/BS (%)	50.2±4.5	40.8±7.7*
MAR (μm^2/day)	0.87±0.17	0.61±0.11**
BFR($\mu m^3/\mu m^2$/day)	0.33±0.07	0.24±0.04*
**Static Histomorphometry (Male, *n* = 4)**		
Oc.N/BS (1/mm)	3.77±1.41	3.35±0.60
Ob.N/BS (1/mm)	3.7±0.4	1.7±0.7**

Tb.BV/TV, trabecular bone volume/tissue volume; Tb.N, trabecular number; Tb.Th, trabecular thickness; Tb.Sp, trabecular spacing; BMD, bone mineral density; Ct.BV/TV, cortical BV/TV; Ct.Th, cortical thickness; T.Ar, total area; MS/BS, mineralizing surface/bone surface; MAR, mineral apposition rate; BFR, bone formation rate; Oc.N/BS, osteoclast number/bone surface; Ob.N/BS, osteoblast number/bone surface. Data represent mean ± SD. $*P < 0.05$, $**P < 0.01$, $***P < 0.001$. Two-tailed Student's paired *t* test.

Supplementary table 3. Bone parameters of DKO mice at 4 months of age

	$Sp7;Gls^{fl/+}Gclc^{fl/+}$	$Sp7;Gls^{fl/fl}Gclc^{fl/+}$	$Sp7;Gls^{fl/+}Gclc^{fl/fl}$	$Sp7;Gls^{fl/fl}Gclc^{fl/fl}$
**Male (*n*)**	10	7	6	8
Tb.BV/TV (%)	15.76±2.45	8.57±1.61***	9.92±1.80**	8.22±1.60***
Tb.N (1/mm)	5.17±0.35	4.34±0.35***	4.62±0.30*	4.36±0.36***
Tb.Th (mm)	0.045±0.004	0.039±0.002**	0.039±0.005*	0.038±0.003***
Tb.Sp (mm)	0.19±0.02	0.23±0.02**	0.22±0.02*	0.23±0.02**
BMD (mg HA/ccm)	214±24	156±24***	184±25*	156±22***
Ct.BV/TV (%)	32.6±0.9	30.7±2.0*	33.4±1.5	31.8±2.2
Ct.Th (mm)	0.199±0.009	0.188±0.017	0.199±0.007	0.181±0.012*
T.Ar (mm^2)	1.89±0.19	1.82±0.29	1.89±0.20	1.62±0.10**
**Female (*n*)**	11	6	7	6
Tb.BV/TV (%)	6.25±2.12	3.94±1.05*	4.58±1.00*	3.72±1.25*
Tb.N (1/mm)	3.45±0.39	3.08±0.42*	3.28±0.20	3.14±0.19
Tb.Th (mm)	0.039±0.003	0.035±0.002*	0.037±0.004	0.035±0.002*
Tb.Sp (mm)	0.30±0.04	0.33±0.04	0.31±0.02	0.32±0.02
BMD (mg HA/ccm)	102±24	87±12*	85±14*	87±18
Ct.BV/TV (%)	31.2±1.5	31.8±2.2	32.4±1.7	30.7±1.4
Ct.Th (mm)	0.173±0.008	0.179±0.016	0.179±0.008	0.170±0.003
T.Ar (mm^2)	1.51±0.14	1.48±0.08	1.52±0.11	1.37±0.03**
**Serum Analysis (Male) (*n*)**	10	7	6	8
P1NP (ng/ml)	93.07±39.98	67.63±27.71*	71.52±34.51*	41.64±15.18**
OCN (ng/ml)	4.62±0.85	3.23±0.67***	3.34±0.73**	3.04±0.78***
CTX-I (ng/ml)	48.01±21.74	40.66±20.51	50.76±22.86	51.80±19.23
**Dynamic Histomorphometry**				
MS/BS (%)	ND	ND	ND	ND
MAR (μm^2/day)	ND	ND	ND	ND
BFR($\mu m^3/\mu m^2$/day)	ND	ND	ND	ND
**Static Histomorphometry (Male, *n* = 4)**				
Oc.N/BS (1/mm)	4.49±0.71	4.26±1.12	4.03±0.66	4.37±0.64
Ob.N/BS (1/mm)	15.02±3.08	5.00±1.12**	6.56±0.63*	4.68±1.57**

Tb.BV/TV, trabecular bone volume/tissue volume; Tb.N, trabecular number; Tb.Th, trabecular thickness; Tb.Sp, trabecular spacing; BMD, bone mineral density; Ct.BV/TV, cortical BV/TV; Ct.Th, cortical thickness; T.Ar, total area; P1NP, procollagen 1 intact N-terminal propeptide; OCN, osteocalcin; CTX-I, C-terminal telopeptide I; MS/BS, mineralizing surface/bone surface; MAR, mineral apposition rate; BFR, bone formation rate; Oc.N/BS, osteoclast number/bone surface; Ob.N/BS, osteoblast number/bone surface. Data represent mean ± SD. $*P < 0.05$, $**P < 0.01$, $***P < 0.001$. All groups were compared with control ($Sp7;Gls^{fl/+}Gclc^{fl/+}$) group. Two-way ANOVA test.

Supplementary table 4. Bone parameters of *Sp7Cre;*$Gls^{fl/fl}$*MitoCat* mice at 4 months of age

	Sp7;$Gls^{fl/+}$	*Sp7;*$Gls^{fl/fl}$	*Sp7;MitoCat*	*Sp7;*$Gls^{fl/fl}$*MitoCat*
**Male (*n*)**	9	6	10	7
Tb.BV/TV (%)	13.97±2.82	8.28±1.52**	14.82±1.84	13.59±2.16
Tb.N (1/mm)	4.69±0.44	4.21±0.56	4.41±0.50	4.15±0.36
Tb.Th (mm)	0.046±0.007	0.039±0.007	0.051±0.005	0.051±0.005
Tb.Sp (mm)	0.21±0.02	0.24±0.03	0.23±0.03	0.24±0.02
BMD (mg HA/ccm)	196±35	139±13**	206±16	192±23
Ct.BV/TV (%)	30.8±2.7	29.0±4.5	32.2±2.2	31.9±2.4
Ct.Th (mm)	0.192±0.023	0.157±0.025*	0.201±0.015	0.203±0.019
T.Ar (mm^2)	2.09±0.24	1.64±0.13**	2.28±0.30	2.20±0.16
**Female (*n*)**	9	7	6	6
Tb.BV/TV (%)	6.38±1.11	3.98±0.74***	5.78±1.29	5.85±0.96
Tb.N (1/mm)	3.51±0.37	3.02±0.19*	3.29±0.31	3.49±0.26
Tb.Th (mm)	0.043±0.005	0.039±0.003	0.041±0.004	0.039±0.002
Tb.Sp (mm)	0.29±0.03	0.33±0.02*	0.31±0.03	0.29±0.02
BMD (mg HA/ccm)	108±12	84±13**	104±10	97±8
Ct.BV/TV (%)	31.5±1.7	30.0±1.5	31.6±2.1	28.8±0.9
Ct.Th (mm)	0.179±0.011	0.170±0.009	0.177±0.011	0.163±0.003
T.Ar (mm^2)	1.59±0.16	1.58±0.13	1.62±0.21	1.57±0.14
**Serum Analysis (Male) (*n*)**	9	6	10	7
P1NP (ng/ml)	67.64±25.82	40.98±14.21*	74.55±24.06	65.81±16.50
OCN (ng/ml)	2.32±0.73	1.60±0.22*	2.09±0.54	2.57±0.77
CTX-I (ng/ml)	29.94±15.33	36.61±18.58	47.53±26.78	34.48±20.65
**Dynamic Histomorphometry (Male, *n* = 4)**				
MS/BS (%)	35.3±4.4	23.8±4.7**	37.5±4.9	37.9±4.3
MAR (μm^2/day)	2.75±0.30	1.20±0.19***	2.83±0.54	2.71±0.42
BFR($\mu m^3/\mu m^2$/day)	0.97±0.17	0.29±0.08***	1.07±0.32	1.02±0.11
**Static Histomorphometry (Male, *n* = 4)**				
Ob.N/BS (1/mm)	21.05±6.20	6.59±2.71**	19.29±3.75	22.03±2.79

Tb.BV/TV, trabecular bone volume/tissue volume; Tb.N, trabecular number; Tb.Th, trabecular thickness; Tb.Sp, trabecular spacing; BMD, bone mineral density; Ct.BV/TV, cortical BV/TV; Ct.Th, cortical thickness; T.Ar, total area; P1NP, procollagen 1 intact N-terminal propeptide; OCN, osteocalcin; CTX-I, C-terminal telopeptide I; MS/BS, mineralizing surface/bone surface; MAR, mineral apposition rate; BFR, bone formation rate; Ob.N/BS, osteoblast number/bone surface. Data represent mean ± SD. *$P < 0.05$, **$P < 0.01$, ***$P < 0.001$. All groups were compared with control (*Sp7;*$Gls^{fl/+}$) group. Two-way ANOVA test.

Supplementary table 5. sgRNA targeting sequences

sgRNA symbol	Sequence
**GCLC.G3**	TAGTGGCCAGCTGATCATAANGG
**GCLC.G4**	TACATGATCGAAGGAACGCCNGG
**GCLC.G7**	TGCTTGTTTATGGCTTCATCNGG
**GCLC.G14**	TCTTGCCTCAGATATGCTGCNGG
**GCLC.G19**	TCAGACATCGTTCCTCCGTANGG
**GCLM.G6**	GATTTATCTTCTCCACTGCANGG
**GCLM.G8**	ACCGGGAACCTGCTCAACTGNGG
**GCLM.G13**	TGGACGGGCACTTTTTTCGCNGG
**GCLM.G21**	GCAGGGTGCTGGCCCGCGCCNGG
**MS344.GLS.G5**	ATATAACTCATCGATGTGTGNGG
**MS344.GLS.G3**	GTGCTAAAAAGCAGTCTGGANGG
**MS345.GLS.G3**	CAAATTCAGTCCTGATTTGTNGG
**MS346.GLS.G14**	ATATTTCAGGGGTTTTACACNGG
**MS346.GLS.G1**	TGCAATTGCTGTTAATGACCNGG
**SP498.MCHERRY.G17**	CAAGTAGTCGGGGATGTCGGNGG
**SP498.MCHERRY.G19**	AGTAGTCGGGGATGTCGGCGNGG
**SP499.LUC.G3**	CAATTCTTTATGCCGGTGTTNGG
**SP399.LUC.G4**	GTGTTGGGCGCGTTATTTATNGG

Supplementary table 6. qPCR primer sequences

Gene symbol	Forward	Reverse
Gclc	GGGGTGACGAGGTGGAGTA	GTTGGGGTTTGTCCTCTCCC
Gclm	AGGAGCTTCGGGACTGTATCC	GGGACATGGTGCATTCCAAAA
Gss	CAAAGCAGGCCATAGACAGGG	AAAAGCGTGAATGGGGCATAC
Gpx3	CCTTTTAAGCAGTATGCAGGCA	CAAGCCAAATGGCCCAAGTT
Gst	CTCAGGCAGCTCATGGACAAT	GTTATCCTCTGGAATGCGGTC
Gsr	GACACCTCTTCCTTCGACTACC	CCCAGCTTGTGACTCTCCAC
Gls	AGGGTGAAGTCGGTGATAAAC	GGGCTGTTCTGGAGTCATAAT
Runx2	CCAACCGAGTCATTTAAGGCT	GCTCACGTCGCTCATCTTG
Sp7	ATGGCGTCCTCTCTGCTTG	TGAAAGGTCAGCGTATGGCTT
Alpl	CCAACTCTTTTGTGCCAGAGA	GGCTACATTGGTGTTGAGCTTTT
Col1a1	GCTCCTCTTAGGGGCCACT	CCACGTCTCACCATTGGGG
Ibsp	CAGAGGAGGCAAGCGTCACT	GCTGTCTGGGTGCCAACACT
Bglap	CAGCGGCCCTGAGTCTGA	GCCGGAGTCTGTTCACTACCTTA
p16	CGCAGGTTCTTGGTCACTGT	TGTTCACGAAAGCCAGAGCG
p21	CCTGGTGATGTCCGACCTG	CCATGAGCGCATCGCAATC
p53	GTCACAGCACATGACGGAGG	TCTTCCAGATGCTCGGGATAC
Il6	TAGTCCTTCCTACCCCAATTTCC	TTGGTCCTTAGCCACTCCTTC
Fas	TATCAAGGAGGCCCATTTTGC	TGTTTCCACTTCTAAACCATGCT
Actb	AGATGTGGATCAGCAAGCAG	GCGCAAGTTAGGTTTTGTCA
